# Whole-transcriptome analysis of atrophic ovaries in broody chickens reveals regulatory pathways associated with proliferation and apoptosis

**DOI:** 10.1038/s41598-018-25103-6

**Published:** 2018-05-08

**Authors:** Lingbin Liu, Qihai Xiao, Elizabeth R. Gilbert, Zhifu Cui, Xiaoling Zhao, Yan Wang, Huadong Yin, Diyan Li, Haihan Zhang, Qing Zhu

**Affiliations:** 1 Farm Animal Genetic Resources Exploration and Innovation Key Laboratory of Sichuan Province, Sichuan Agricultural University, Chengdu Campus, 611130 Sichuan Province, China; 2 Department of Animal and Poultry Sciences, Virginia Tech, Blacksburg, 24061 Virginia USA

## Abstract

Broodiness in laying hens results in atrophy of the ovary and consequently decreases productivity. However, the regulatory mechanisms that drive ovary development remain elusive. Thus, we collected atrophic ovaries (AO) from 380-day-old broody chickens (BC) and normal ovaries (NO) from even-aged egg-laying hens (EH) for RNA sequencing. We identified 3,480 protein-coding transcripts that were differentially expressed (DE), including 1,719 that were down-regulated and 1,761 that were up-regulated in AO. There were 959 lncRNA transcripts that were DE, including 56 that were down-regulated and 903 that were up-regulated. Among the116 miRNAs that were DE, 79 were down-regulated and 37 were up-regulated in AO. Numerous DE protein-coding transcripts and target genes for miRNAs/lncRNAs were significantly enriched in reproductive processes, cell proliferation, and apoptosis pathways. A miRNA-intersection gene-pathway network was constructed by considering target relationships and correlation of the expression levels between ovary development-related genes and miRNAs. We also constructed a competing endogenous RNA (ceRNA) network by integrating competing relationships between protein-coding genes and lncRNA transcripts, and identified several lncRNA transcripts predicted to regulate the *CASP6*, *CYP1B1*, *GADD45*, *MMP2*, and *SMAS2* genes. In conclusion, we discovered protein-coding genes, miRNAs, and lncRNA transcripts that are candidate regulators of ovary development in broody chickens.

## Introduction

Broodiness is a maternal behavior in hens that is characterized by increased body temperature, reduced food and water intake, frequent nest occupancy, increased incubation of eggs, and cessation of laying, the results of which have major impacts on the poultry industry^1^, as it is common in most domestic fowls^2^. The condition results in atrophy of the ovary and in broody geese, this was associated with the appearance of white follicles (WF) and the absence of small yellow follicles (SYF) and large yellow follicles (LYF)^3,4^, suggesting that there is slow development of WF, no transition of WF into SYF, or direct atresia of SYF. The initiation and maintenance of ovarian atrophy in broody chickens involves a series of phenotypic and physiological changes that are poorly understood at the molecular level^5^, although the endocrine mechanisms and identification of candidate genes have been the focus of much research.

In broody hens, decreased gonadotrophin-releasing hormone (*GnRH*) and increased vasoactive intestinal polypeptide (*VIP*) release from the hypothalamus induced production of prolactin (PRL)^6,7^. Three genes, encoding the anti-mullerian hormone receptor II (*AMHRII*), prolactin receptor (*PRLR*), and estrogen receptor α (*ERα*), were identified as being associated with triggering or maintaining broodiness in geese^8^. Genetic variations in *PRL*, *PRLR*, *VIP* receptors and the dopamine D1 receptor had significant effects on the frequency and duration of broodiness^6,7,9,10^. Reactive oxygen species (ROS) activate autophagy in follicular granulosa cells via the mTOR pathway to regulate broodiness in geese^11^. A reduction in *MAPK* signaling and/or elevation of cAMP signaling enhanced *FSHR* expression and granulosa cell differentiation^12^. Thus, although candidate genes for bird broodiness have been identified, the molecular mechanisms and associated signaling pathways remain poorly understood.

The advent of technology for sequencing RNA transcripts has led to the realization that non-coding RNAs have important functions in development and metabolism. Non-coding RNAs include microRNAs (miRNAs), transfer RNAs (tRNAs), ribosomal RNAs (rRNAs), small interfering RNAs (siRNAs), and long non-coding RNAs (lncRNAs)^13,14^. miRNAs are a class of highly conserved, endogenous, single-stranded, and small non-coding RNA molecules (approximately 18–25 nucleotides in length) that function in post-transcriptional regulation of gene expression through translational repression or target mRNA degradation via binding to their 3′untranslated regions (3′UTRs)^15^. miRNAs are involved in multiple biological processes including cell proliferation, differentiation, apoptosis, organogenesis, and disease pathogenesis^16^. Results from recent studies have demonstrated that miRNAs can control steroidogenesis and regulate proliferation and apoptosis of granulosa cells in the human ovary^17,18^. X-linked miR-503, miR-672, and miR-465 families, which are preferentially expressed in newborn mouse testes and ovaries, participate in pathways associated with folliculogenesis^19^. miR-125b was identified as a highly abundant miRNA at each developmental stage of follicles that decreased during *in vitro* luteinization of theca cells^20^. Thus, miRNAs play important roles in ovarian development.

LncRNAs are non-protein-coding transcripts ranging from 0.2 kb to 100 kb in length that tend to be poorly conserved among species, and display low to moderate expression in a tissue- and time-specific manner^21^. Based on their location in the genome, lncRNA can be divided into five categories: antisense lncRNA, intronic transcript, intergenic lncRNA, promoter-associated lncRNA, and UTR-associated lncRNA^22^. lncRNAs have a wide range of functions in cellular and developmental processes including genomic imprinting, chromatin remodeling, histone modification, transcriptional and post-transcriptional regulation, apoptosis, and cell cycle regulation^23–25^. These studies focused on humans and typical laboratory models (e.g. rat, mouse and nematodes)^26–29^, and information concerning other species is scarce, especially those of agricultural relevance. There are no reports of the involvement of lncRNAs in ovary atrophy of broody hens.

The transcriptome is the complete set of transcripts in a population of cells or a single cell, including mRNA, miRNA, and lncRNA, etc^30^. In contrast with the genome, the transcriptome is spatiotemporally regulated and reflects gene expression under certain physiological conditions or developmental stages^23,24^. RNA-seq, as a next-generation sequencing technology, is a highly sensitive method for whole transcriptome analysis^31^. To date, the approach has been applied to chicken in studying the skeletal muscle, adipose tissue, liver, spleen, pituitary, hypothalamus, and ovary^16,23,24,31–33^. Such data are meaningful in biomarker discovery and identifying pathways that govern growth and developmental processes, metabolism, and reproductive biology. The objective of this experiment was thus to use RNA-seq to identify transcripts and pathways that are associated with atrophy of the chicken ovary.

## Results

### Comparison of ovarian morphological and histological characteristics, and plasma hormones

**Figure 1:**
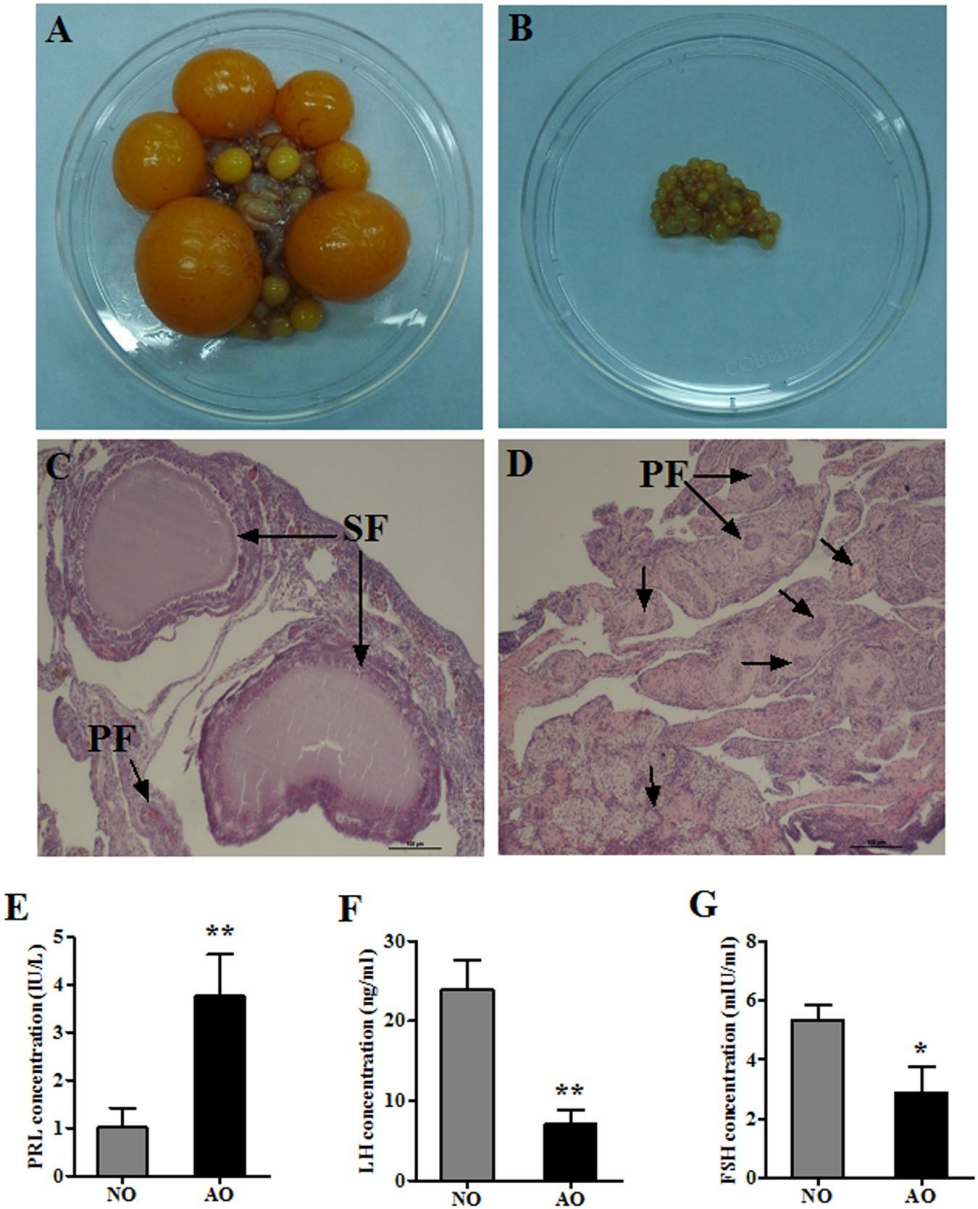
Ovarian morphological and histological characteristics and plasma hormones in egg-laying and broody chickens. (**A**) Morphological characteristics in the normal ovary (NO) of egg-laying hens (EH) and (**B**) the atrophic ovary (AO) of broody chickens (BC). (**C**) Histological characteristics in NO and (**D**) AO (HE staining at 100×); SF represents secondary follicles and PF is the primary follicles. (**E**–**G**) Plasma concentrations of prolactin (PRL), luteinizing hormone (LH) and follicle stimulating hormone (FSH) in EH and BC; results are expressed as means ± standard deviation (n = 6); *P < 0.05; **P < 0.01.

Egg-laying hens had plump ovaries with many visible follicles and a gradually increasing volume, whereas ovaries of broody chickens showed obvious atrophy with visible characteristics (Fig. 1A,B). Ovary weights and ratio (Ovary weight/Body weight *100%) and stroma weights of broody chickens were significantly lower than those of egg-laying hens (P < 0.05), and LYF and SYF were not observed in broody hens (Table S2). Observation under the light microscope revealed an unconsolidated ovary with many primary follicles (one layer of cuboidal granulosa cells, PFs) and secondary follicles (two to six layers of granulosa cells, SFs) in egg-laying hens (Fig. 1C), while the broody chickens had numerous PFs but few SFs within the more compact ovary (Fig. 1D). Plasma concentrations of PRL, LH, and FSH were different between egg-laying and broody chickens (P < 0.05) (Fig. 1E,F and G, respectively).

### Overview of RNA-sequencing

To obtain a global view of the chicken ovary transcriptome and identify the protein-coding and lncRNA transcripts related to AO of BC, six strand-specific libraries were constructed and sequenced, resulting in a total of 508.93 M (million) raw reads, which yielded 63.3 Gb (giga bases) of raw data, and an average of 84.8 M of raw reads was obtained per library. And about 98.7% (502.42 M) of raw reads passed initial quality thresholds and were deemed as clean reads for the subsequent analyses, where an average clean reads was 83.74 M per library (Tables S3 and S4). We identified 58,175 reliable transcripts (Fig. S1A) including 15,952 known and 42,223 novel transcripts. Approximately 75% of novel transcripts in each library showed a high coverage with at least 90% for transcript being covered by reads (Fig. S2).

In the all sequencing libraries, we identified 49,461 protein-coding transcripts including 15,952 known and 33,509 novel protein-coding transcripts. After transcripts abundances were quantified by FPKM (Fragments Per Kilobase of transcript per Million mapped reads), the average expression level of novel protein-coding transcripts (4.33) is about one-sixth of known protein-coding transcripts (24.81). 49,290 protein-coding transcripts were detected in the whole transcriptome of AO, and 49,163 protein-coding transcripts were detected in NO. A total of 48,992 protein-coding transcripts were co-expressed in NO and AO, while 171 and 298 protein-coding transcripts were specifically expressed in NO and AO, respectively (Fig. S3A). All protein-coding transcripts corresponded to 14,447 protein-coding genes, with an average of 3.42 transcripts per gene locus. 33,509 novel protein-coding transcripts corresponded to 11,504 protein-coding genes, with an average of 2.91 transcripts per gene locus. 79.63% protein-coding genes that had novel transcripts were identified. We also detected a large number of alternative splicing events in six sequencing libraries, which averaged 54,010 per library. The distribution of alternative splicing events in all libraries is similar, with three types of events being prevalent including TSS (Alternative 5′ first exon), TTS (Alternative 3′ last exon), and SKIP (Exon skipping) (Fig. S4).

**Figure 2:**
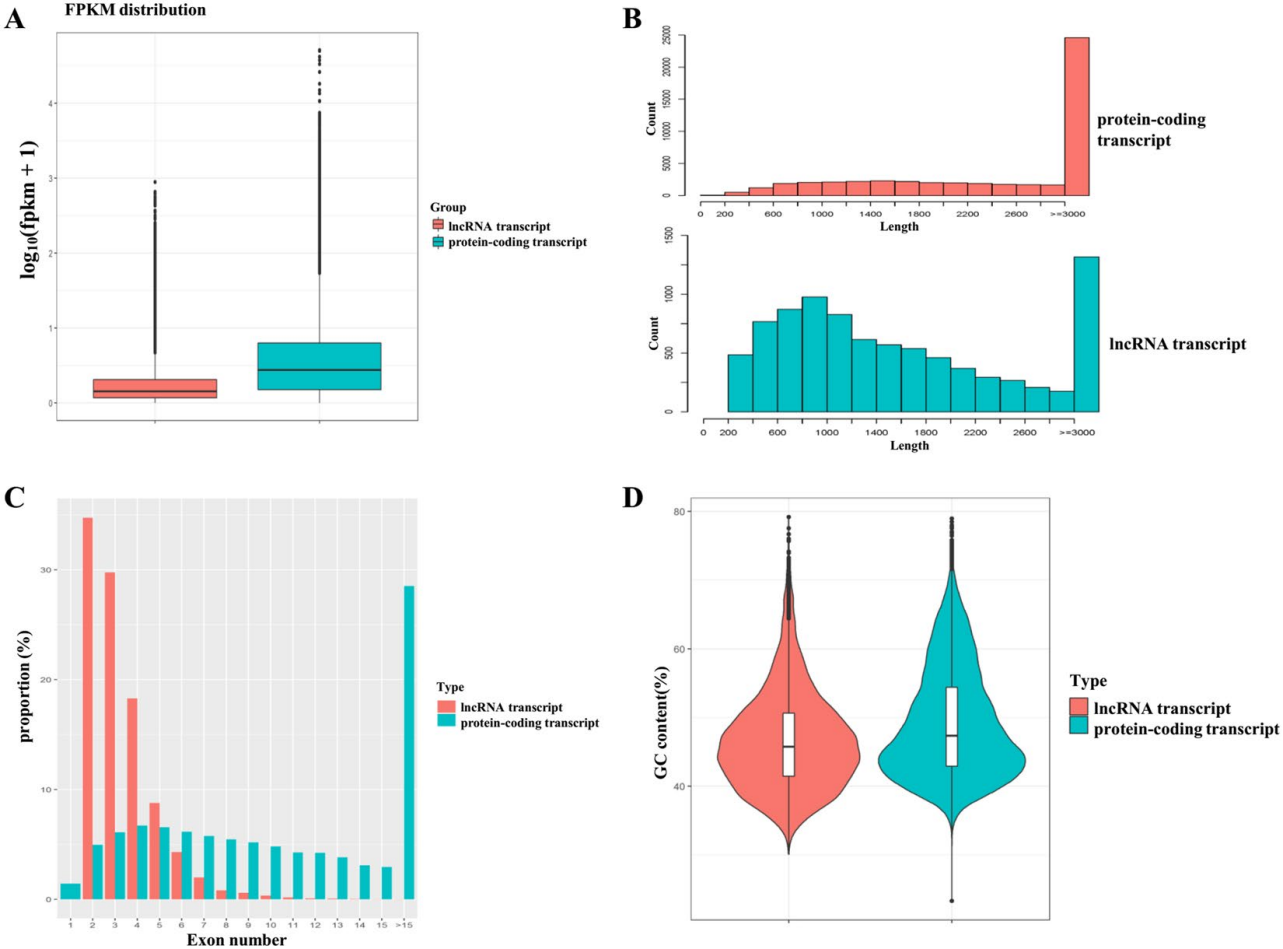
Overview of RNA sequencing in the chicken ovary. (**A**) The boxplot of FPKM (Fragment Per Kilobase of transcript per Million mapped reads) for protein-coding and lncRNA transcripts; (**B**) The distribution of read lengths for protein-coding and lncRNA transcripts. (**C**) The proportions of exons’ number per transcript for protein-coding and lncRNA transcripts. (**D**) The GC content of protein-coding and lncRNA transcripts.

In total there were 8,714 lncRNA transcripts. 8,684 lncRNA transcripts were detected in the whole transcriptome of AO, and 8,561 lncRNA transcripts were detected in NO. A total of 8,531 lncRNA transcripts were co-expressed in NO and AO, while 30 and 153 lncRNA transcripts were specifically expressed in NO and AO, respectively (Fig. S3B). The lncRNA transcripts corresponded to 4,273 lncRNA genes, with an average of 2.04 transcripts per gene locus. A boxplot of FPKM, histogram of lengths, proportion of exon number per transcript, and GC content for protein-coding and lncRNA transcripts are shown in Fig. 2. The average expression level of lncRNA transcripts (2.12) was lower than protein-coding transcripts (11.24) (Fig. 2A). Protein-coding transcripts with an average length of 3,736 bp and 9.67 exons were longer than the lncRNA transcripts, which averaged 1,853 bp and 3.36 exons (Fig. 2A,B). The average GC content of protein-coding and lncRNA transcripts was 48.46% and 46.66%, respectively (Fig. 2D). These findings that are in agreement with those of previous studies^23,24,34^. The lncRNA transcripts included 7,241 intergenic (82.9%) and 846 antisense (9.7%) (Fig. S5A). Most of the lncRNA transcripts were distributed in chromosomes 1–6 (Fig. S5B).

### Analysis of differentially expressed protein-coding transcripts

**Figure 3:**
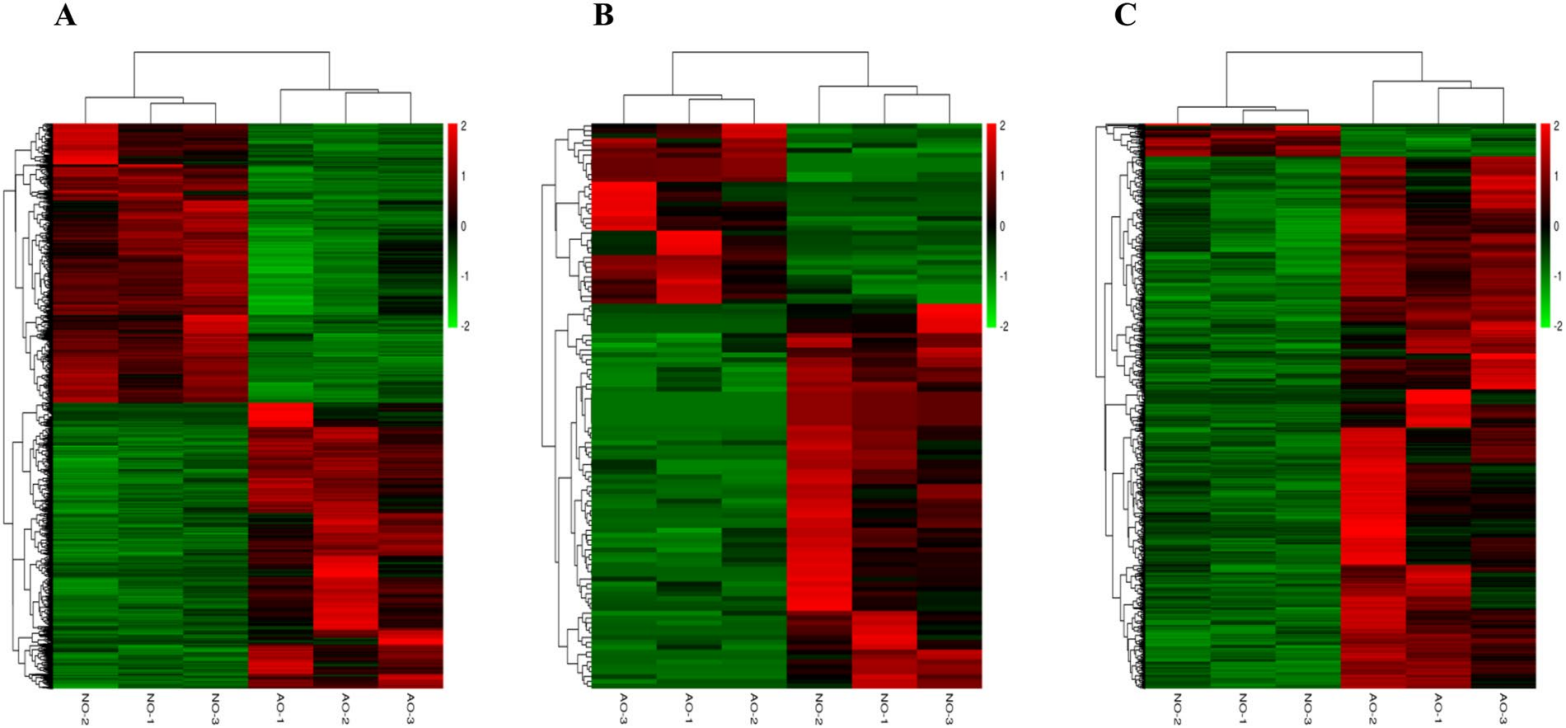
The Hierarchical Cluster Analysis of differentially expressed protein-coding transcripts (**A**), miRNAs (**B**), and lncRNA transcripts (**C**).

**Figure 4:**
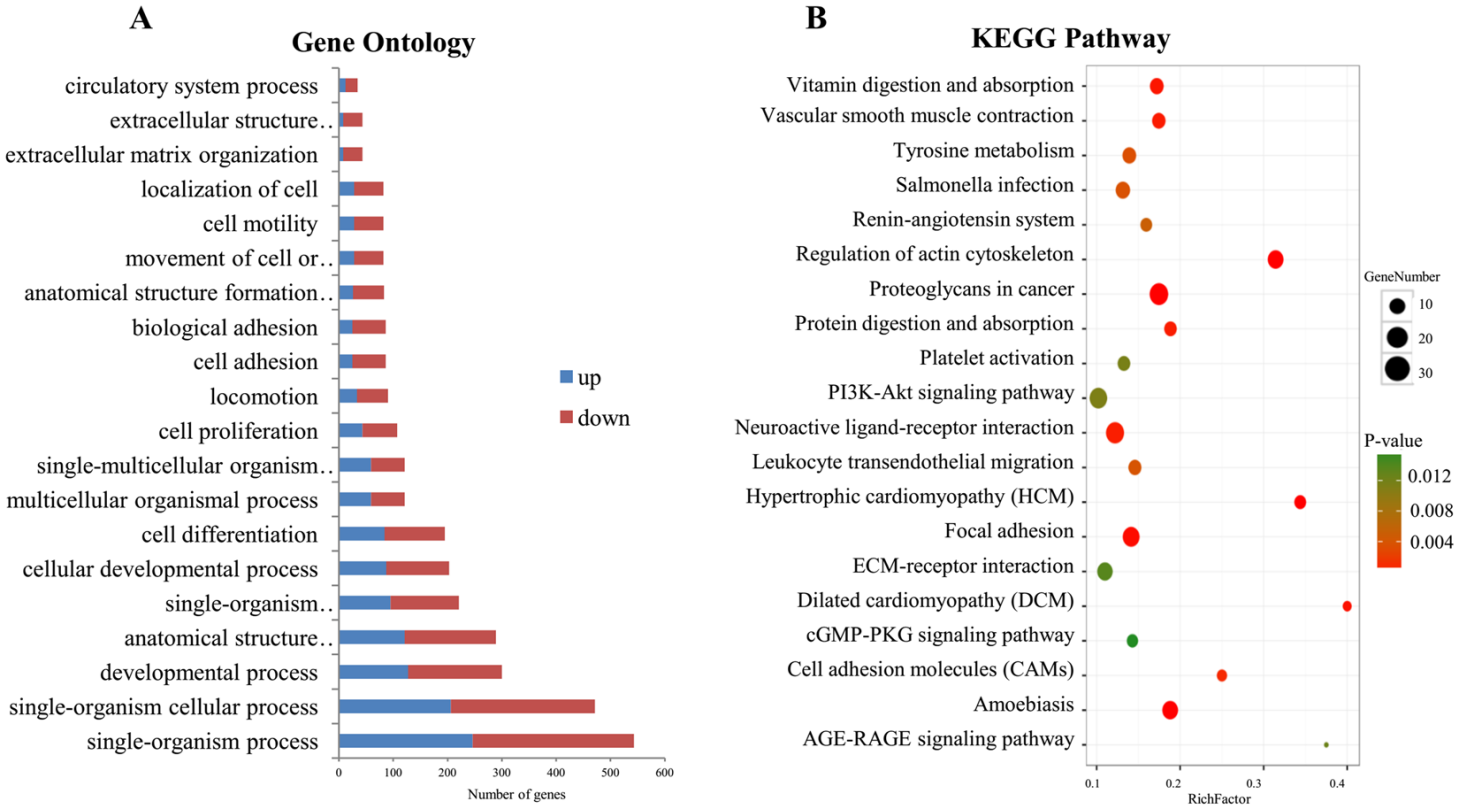
GO/pathway analysis of differentially expressed protein-coding transcripts. (**A**) Top 20 significantly changed GOs of protein-coding transcripts in biological processes; blue and kermesinus show up- and down-regulation in the atrophic ovary of broody chickens, respectively. (**B**) Top 20 significantly changed pathways associated with protein-coding transcripts.

Overall, 3,480 significantly differentially expressed protein-coding transcripts (DEGs) including 1,719 down-regulated (49.4%) and 1,761 up-regulated (50.6%) were discovered in the AO of BC (Fig. 3A). We carried out gene ontology (GO) and pathway enrichment analysis to identify biological functions of DEGs. Among these, the most important cellular components involved the extracellular region, extracellular matrix, extracellular space, membrane, cell periphery, and Golgi apparatus. The molecular functions consisted of cytoskeletal protein binding, structural molecule activity, peptidase activity, and ion binding. In biological processes, the important GO terms were developmental process, anatomical structure formation involved in anatomical structure development, cell differentiation, single-organism process, cell proliferation, anatomical structure morphogenesis, and reproduction (Fig. 4A and Table S7). In the KEGG pathway analysis, several genes were enriched from signal transductions pathways including the PI3K-Akt, MAPK, TGF-beta, cGMP-PKG, and Hippo signaling pathways (Fig. 4B and Table S8). In the enrichment of signal pathways, we identified progesterone-mediated oocyte maturation, ovarian steroidogenesis, and the gonadotropin-releasing hormone (*GnRH*), *PRL*, oxytocin, and estrogen signaling pathways. These belong to the reproductive endocrine system that is important for ovarian development and regulation of broodiness in chicken^31^. There were 17 genes were associated with the above pathways, including *CYP1B1*, *DBH*, *GABBR2*, *GNA11*, *GUCY1A2*, *HSD3B2*, *HSP90*, *INHA*, *INHB*, *MMP2*, *MYL9*, *MYLK*, *OXTR*, *PRKAB2*, *ROCK1*, *ROCK2*, and *TH* (Table S9). We also detected involvement of a few interesting pathways involved in cell growth and death, such as the cell cycle pathway, p53 signaling pathway, and apoptosis. Others have shown that these pathways were associated with follicular granulosa cell growth, proliferation, survival and apoptosis^16,31,35–40^. The genes associated with these pathways included *CAPN2*, *CASP6*, *CASP7*, *CDC20*, *CDC25A*, *GADD4*5, *MAPK11*, *ORC2*, *RIPK1*, *SMAD2*, *SKP2*, *THBS1*, and *WEE2* (Table S9).

### Overview of small-RNA sequencing

**Figure 5:**
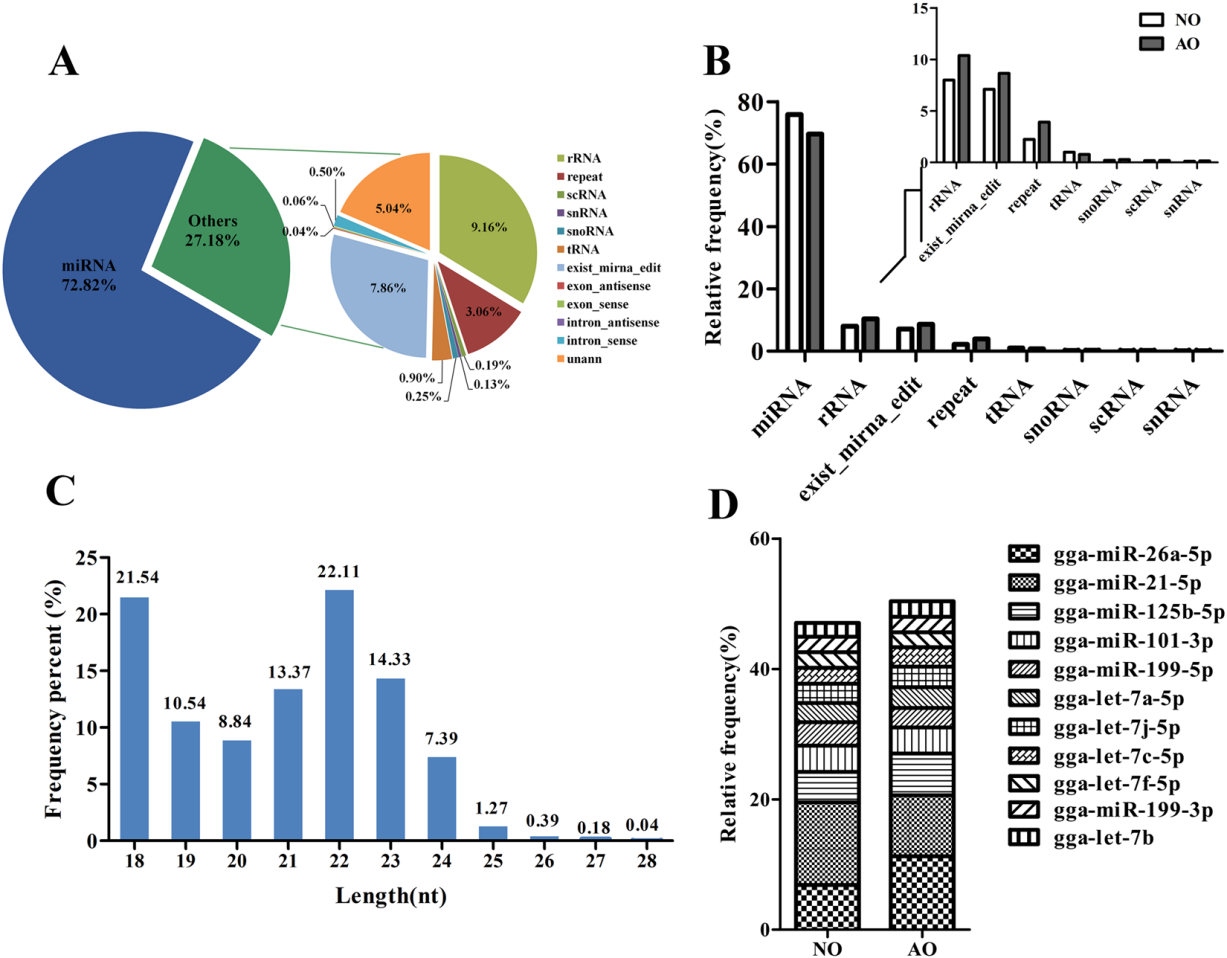
Overview of small RNA sequencing in the chicken ovary. (**A**) Portions of small RNA types in the clean reads. The percent of miRNA is approximately 73%, and the other 28% included rRNA, scRNA, snRNA, snoRNA, tRNA, simple repeats, exon sense/antisense and intron sense/antisense. (**B**) Relative frequency of different types of small RNAs in the atrophic ovary (AO) and normal ovary (NO). (**C**) Size distribution of all miRNAs. The X-axis depicts their length (nt), and the Y-axis represents frequency (%). (**D**) The relative proportion of the top 10 miRNAs in the total amount of miRNA.

As shown in Table S8, approximately 71.33 Mb clean reads were obtained in six libraries, representing a high ratio of clean reads, >96.5% (Table S10). After alignment with small RNAs in GenBank, Rfam and the reference genome, we identified 72.82% mature miRNAs, and the remaining small RNAs (27.18%) included rRNA, scRNA, snRNA, snoRNA, tRNA as well as exist-miRNA-edit (Fig. 5A). Exist-miRNA-edit, which represents the set of miRNA containing mirSNPs (miRNA-related single nucleotide polymorphisms), was up-regulated in AO (Fig. 5B). mirSNPs exert their effect by preventing the biosynthesis of miRNA, and some studies have reported that mirSNPs are significantly associated with disease^41,42^. We identified a total of 2,827 miRNAs in six small RNA libraries (Fig. S1B), and the length of most miRNAs were 18~24 nucleotides (Fig. 5C). Total 2,235 and 2,369 miRNAs were detected from AO and NO, respectively. A total of 1,777 miRNA were co-expressed in NO and AO, while 592 and 458 miRNA were specifically expressed in NO and AO, respectively (Fig. S3C). Ten mature miRNAs with the highest expression comprised approximately 50% of all miRNAs, showing a relatively abundant distribution (Fig. 5D), while miR-21, miR-26a, miR-125b, miR-101, and miR-199 were the most abundant miRNAs overall, together accounting for 33% of the total.

### Analysis of differentially expressed miRNAs

There were 116 significantly differentially expressed miRNAs (DEMs) including 37 that were up-regulated (31.9%) and 79 that were down-regulated (68.1%) in AO of BC (Fig. 3B). A total of 9,081 target protein-coding genes for DEMs were identified. Among them, 508 genes had transcripts identified as DEGs and also significantly negatively correlated with miRNA expression, and were assigned as intersection genes, which were more likely to be predicted miRNA target genes^32^. We performed GO and pathway enrichment analysis of intersection genes to identify biological functions of miRNAs. These genes were mainly associated with biological processes including anatomical structure development, single-organism process, cell differentiation, cell proliferation, localization, and reproduction (Fig. S6A). The KEGG results revealed that the enriched pathways involved PI3K-Akt signaling, cGMP - PKG signaling, FoxO signaling, cell cycle, Hippo signaling, MAPK signaling, apoptosis, TGF-beta signaling, estrogen signaling, and oxytocin signaling pathways (Fig. S6B).

### Construction of miRNA-gene-pathway relationship network

**Figure 6:**
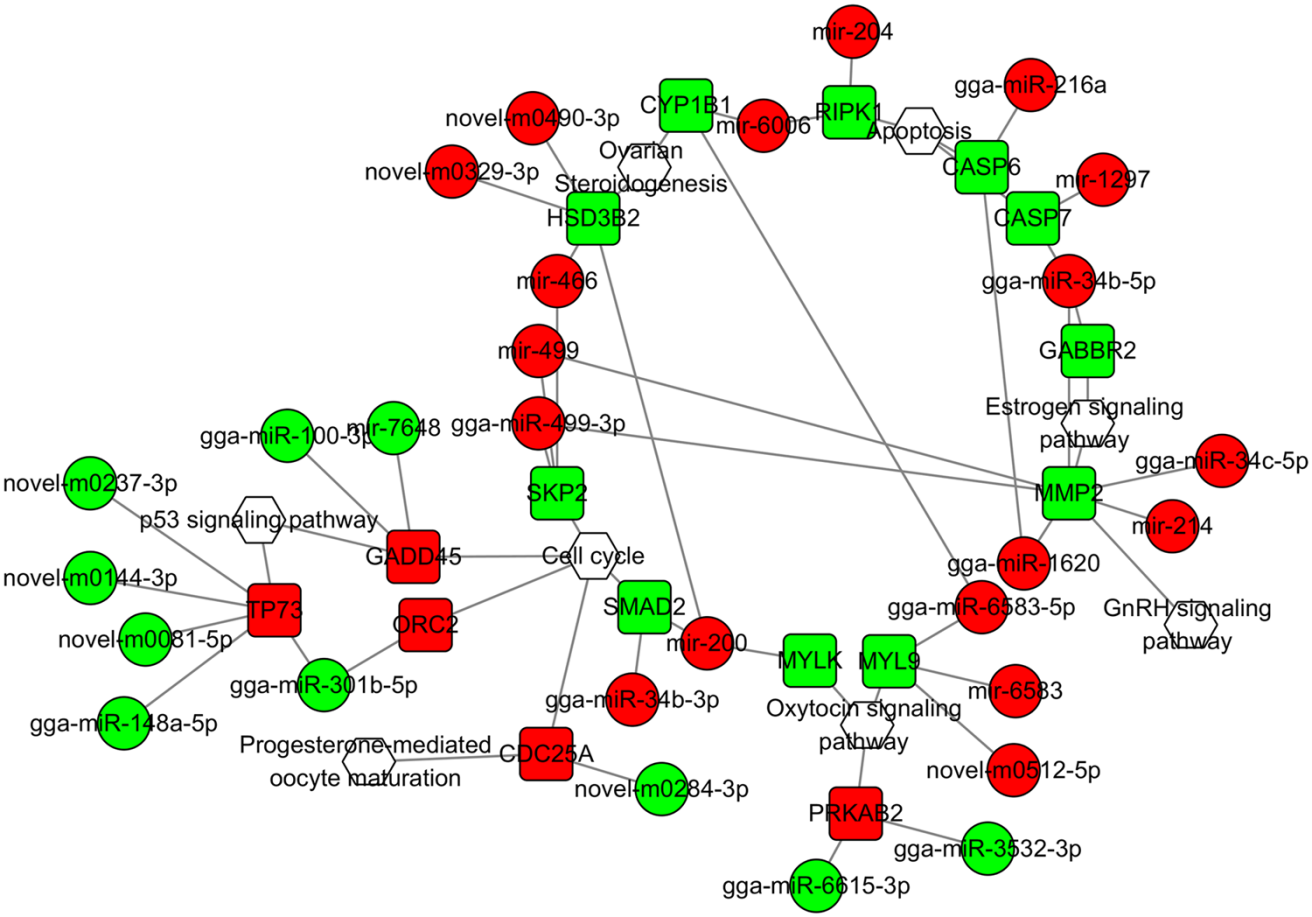
The miRNA-gene-pathway network between sixteen intersection genes, and their corresponding pathways and differentially expressed miRNAs. Hexagon, round rectangle and ellipse indicate pathway, gene and miRNA, respectively. Red and green mean up- and down-regulation, respectively.

Among the intersection genes, 16, involved in reproductive endocrine system, and cell growth and death pathways, corresponded with 31 DEMs (Table 1). Cell division cycle 25 A (*CDC25A*), growth arrest and DNA damage-inducible 45 (*GADD45*), and matrix metallopeptidase 2 (*MMP2*) genes play roles in multiple pathways. To further understand and visualize the interactions and investigate the function of corresponding DEMs, an miRNA-gene-pathway network was constructed (Fig. 6) using the data from Table 1. Through the interaction analysis, we identified potential functions of a few miRNAs in the ovary: (1) gga-miR-34c, gga-miR-3532, gga-miR-6583, and gga-miR-6615 were closely associated with reproductive processes and ovarian steroidogenesis; (2) gga-miR-100, gga-miR-148a, gga-miR-216a, and gga-miR-301b may play important roles in cell proliferation and apoptosis; (3) gga-miR-1620, gga-miR-34b, and gga-miR-499 were associated with multiple pathways including reproductive processes, cell cycle, p53 pathway, and apoptosis.

**Table 1:** Sixteen intersection genes and their corresponding pathways and differentially expressed miRNAs^*^.

Differentially expressed genes	Pathway	Related differentially expressed miRNA
*CASP6*	Apoptosis	gga-miR-1620, gga-miR-216a
*CASP7*	Apoptosis	gga-miR-34b-5p, miR-1297
*CDC25A*	Progesterone-mediated oocyte maturation, Cell cycle	novel-m0284-3p
*CYP1B1*	Ovarian steroidogenesis	gga-miR-6583-5p, miR-6006
*GABBR*	Estrogen signaling pathway	gga-miR-34b-5p
*GADD45*	Cell cycle, p53 signaling pathway	gga-miR-100-3p, miR-7648
*HSD3B2*	Ovarian steroidogenesis	miR-200, miR-466, novel-m0329-3p, novel-m0490-3p
*SMAD2*	Cell cycle	gga-miR-34b-3p, miR-200
*MMP2*	Estrogen signaling pathway, GnRH signaling pathway	gga-miR-1620, gga-miR-34b-5p, gga-miR-34c-5p, gga-miR-499-3p, mirR-214, miR-499
*MYL9*	Oxytocin signaling pathway	gga-miR-6583-5p, miR-6583, novel-m0512-5p
*MYLK*	Oxytocin signaling pathway	miR-200
*ORC2*	Cell cycle	gga-miR-301b-5p
*PRKAB2*	Oxytocin signaling pathway	gga-miR-3532-3p, gga-miR-6615-3p
*RIPK1*	Apoptosis	miR-204, miR-6006
*SKP2*	Cell cycle	gga-miR-499-3p, miR-466, miR-499
*TP73*	p53 signaling pathway	gga-miR-148a-5p, gga-miR-301b-5p, novel-m0081-5p, novel-m0144-3p, novel-m0237-3p

^*^Relative abundance of the gene or miRNA when comparing the performance between the broody ovary library and the egg-laying ovary library sequenced by deep sequencing.

### Analysis of differentially expressed lncRNA

We identified 959 lncRNA transcripts that were differentially expressed between the two kinds of ovaries, including 56 that were down-regulated (5.84%) and 903 that were up-regulated (94.16%) in AO (Fig. 3C). Through cis- and trans-regulatory relationship analysis, we detected 1,001 potential target protein-coding genes of the differential lncRNA transcripts. Functional analysis showed that these target genes were significantly enriched in 6,267 GO terms (959 under molecular function, 570 under cellular component, and 4,738 under biological process), and many terms were related to morphogenesis, meiosis, signal transduction, and gene expression. For example, the top 20 terms of biological process involved positive regulation of meiosis I, negative regulation of anion transmembrane transport, labyrinthine layer morphogenesis, embryonic placenta morphogenesis, regulation of activation of Janus/JAK2 kinase activity, and termination of G-protein coupled receptor signaling pathway (Fig. S7A). In addition, the target genes were enriched in 270 pathways, several of which were associated with ovarian development including PI3K-Akt signaling, MAPK signaling, TGF-beta signaling, dopaminergic synapse, cGMP - PKG signaling, Hippo signaling, Wnt signaling, oxytocin signaling, GnRH signaling, estrogen signaling, and oocyte meiosis pathways (Fig. S7B). These results indicated that lncRNAs took part in several biological processes in the chicken ovary.

### Construction of competing endogenous RNA (ceRNA) network

**Figure 7:**
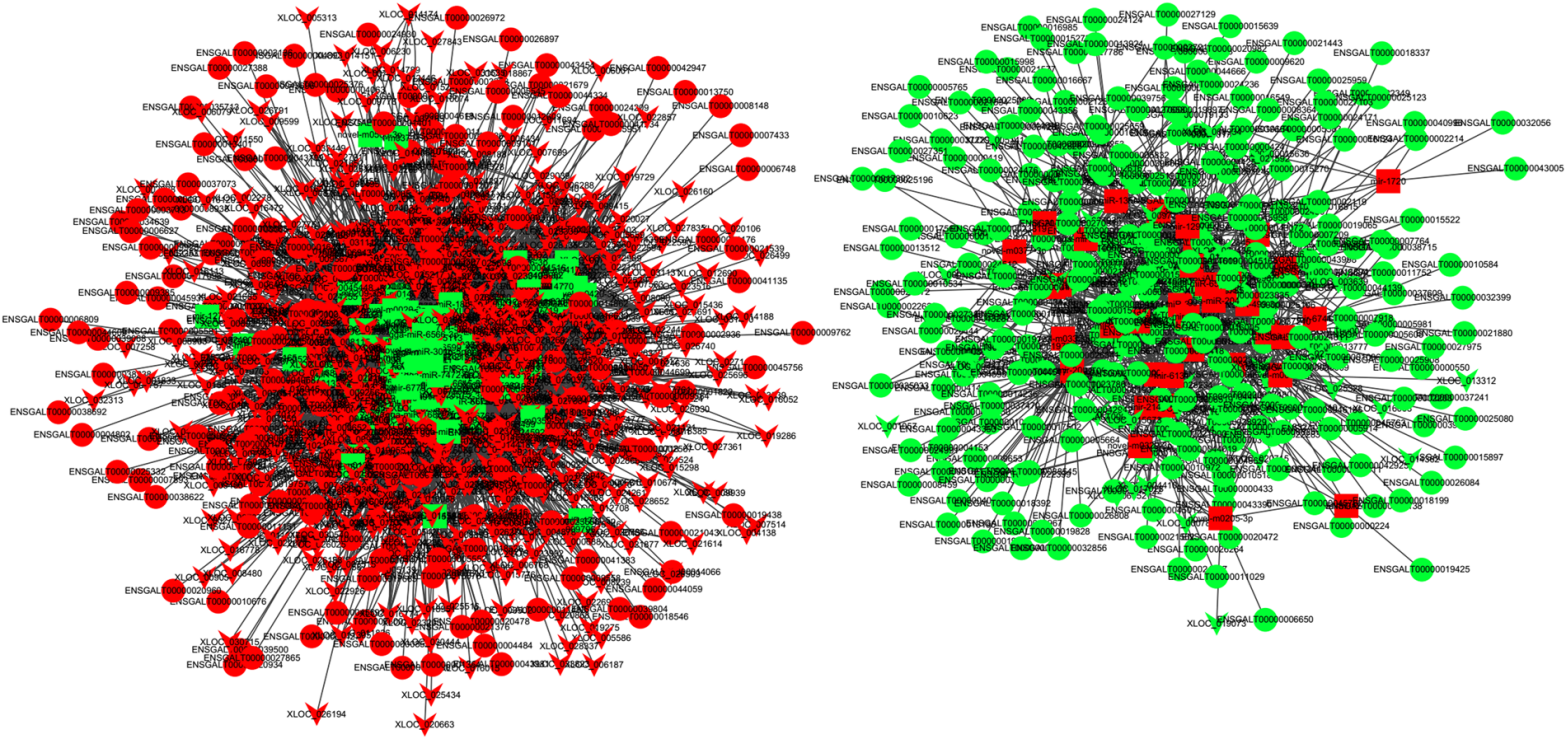
An overview of the competing endogenous RNA (ceRNA) network. Rectangle, ellipse and V indicate miRNA, protein-coding transcript and lncRNA transcript, respectively. Green and red indicate down- and up-regulation, respectively.

Based on the data for differentially expressed protein-coding transcripts, miRNA, and lncRNA transcripts, we used three softwares RNAhybrid(v2.1.2) + svm_light(v6.01), Miranda(v3.3a) and TargetScan(Version:7.0) to identify biological targets of each miRNA from the protein-coding and lncRNA transcripts that showed a significantly negative correlation with miRNA expression, subsequently obtained the protein-coding transcript -miRNA and lncRNA transcript-miRNA pairs, then constructed the competing endogenous RNA (ceRNA) network (Fig. 7), which showed up-regulated miRNAs with decreased expression of protein-coding and lncRNA transcripts, or down-regulated miRNAs with overexpression of protein-coding and lncRNA transcripts. The network consisted of 1,228 nodes with an average degree of 8.65, which indicated that the ceRNA network was dense (Fig. S8).

**Figure 8:**
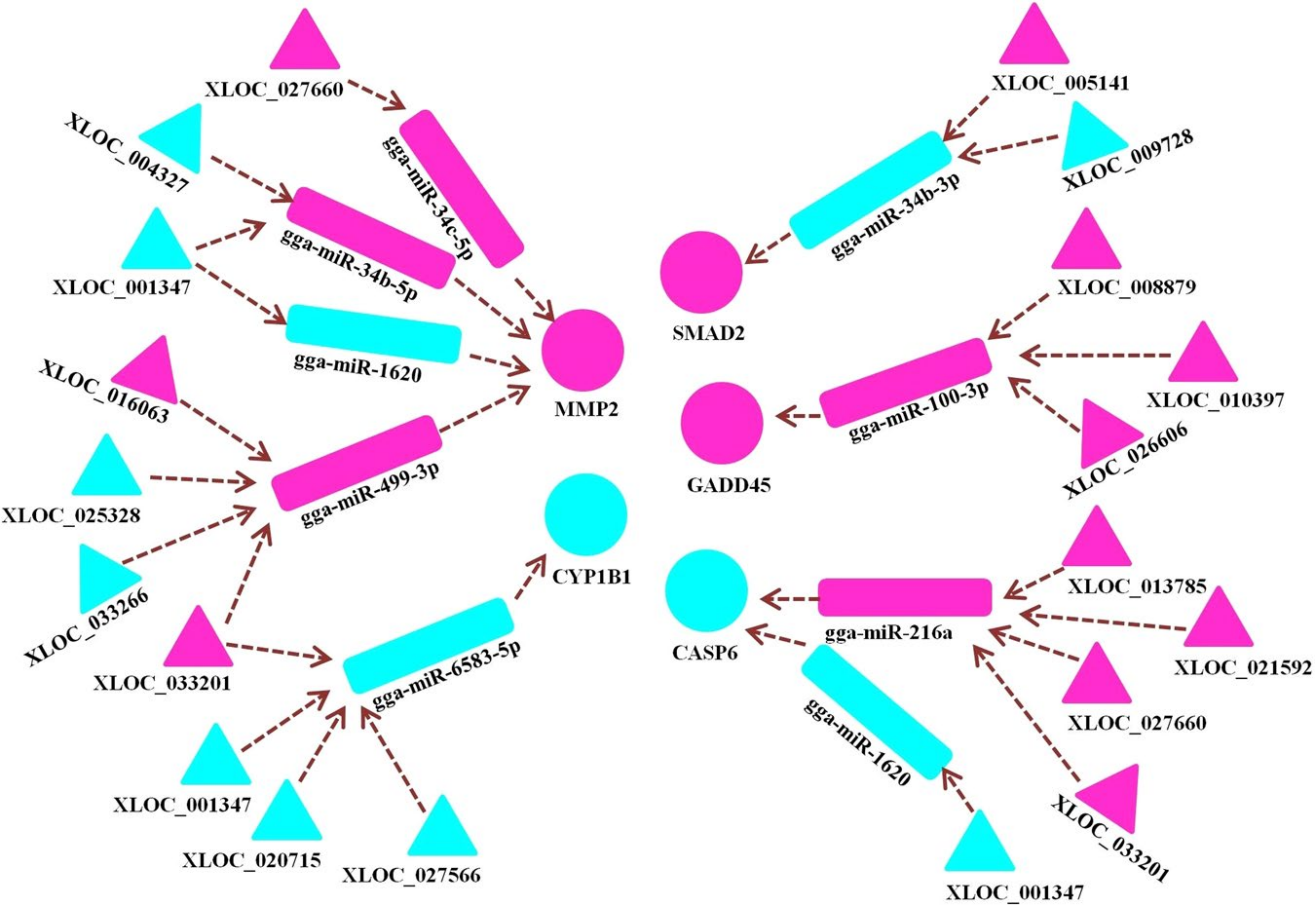
Predicted interaction between lncRNA transcripts and *MMP2*, *CYP1B1*, *SMAD2*, *GADD45*, and *CASP6* genes. Aqua green and pink denote lower and higher expression levels in the atrophic ovary (AO), respectively.

Through combining analysis of the miRNA-gene-pathway and ceRNA networks, interactions between lncRNA transcripts and the reproduction-related *MMP2* and *CYP1B1* genes were predicted (Fig. 8). Highly expressed lncRNA transcripts included XLOC_016063, XLOC_027660, and XLOC_033201, and poorly expressed lncRNA transcripts included XLOC_001347, XLOC_004327, XLOC_020715, XLOC_025328, XLOC_027566, and XLOC_033266. Seven highly expressed and three poorly expressed lncRNA transcripts were also predicted to interact with *SMAD2, GADD45*, and *CASP6* genes, which are strongly associated with cell proliferation and apoptosis. In addition, these interactions referred to 8 miRNAs including gga-miR-34b-3p, gga-miR-34b-5p, gga-miR-34c-5p, gga-miR-100-3p, gga-miR-216a, gga-miR-499-3p, gga-miR-1620, and gga-miR-6583-5p (Fig. 8).

### Validation of RNA-seq data

Five protein-coding genes, five miRNAs, and five lncRNA transcripts from the ceRNA network were selected for validation of the RNA-seq results using real time quantitative PCR (RT-qPCR). The results were consistent with the RNA-seq data (Fig. S9) and a high correlation was detected, with a Pearson’s correlation coefficient of 0.8013 (Fig. S10). The RT-qPCR results of four protein-coding gene-miRNA-lncRNA transcript pairs all showed significant reciprocal expression patterns between miRNA and protein-coding gene and lncRNA transcripts (Fig. S11), consistent with the results of the RNA-seq where miRNAs predominantly function to decrease levels of target protein-coding and lncRNA transcripts.

## Discussion

Broodiness, which is a maternal behavior and instinct in most domestic fowls, reduces laying performance, a major economic concern in the poultry industry^31^. The maintenance of ovarian atrophy in broody chickens is directly related to the broody period length and recovery to egg-laying^2^. To provide a comprehensive view of the transcriptome level changes that occur within the atrophic ovary of broody chickens, whole transcriptome analysis was used to elucidate candidate gene function and their regulatory effectors. In total, 3,480 protein-coding transcripts, 116 miRNAs, and 959 lncRNA transcripts were differentially expressed in the atrophic ovaries of broody chickens.

The hypothalamic pituitary gonadal (HPG) axis mediates ovary development via reproductive endocrine hormones including GnRH, PRL, LH, FSH, oxytocin, estradiol, and progesterone in chickens^31^. In the present study, along with a significant difference in circulating PRL, LH, and FSH, we detected differentially expressed genes relevant to reproductive endocrine hormones in the atrophic ovary of broody chickens. For example, 3β-hydroxy steroid dehydrogenase type 2 (*HSD3B2*) and cytochrome P450 family 1 subfamily B member 1 (*CYP1B1*) genes, which encode crucial enzymes in ovarian steroidogenesis^43^, were decreased in broody chickens. This finding indicates that the broody chicken ovary has weakened steroidogenesis, including synthesis of estradiol and progesterone and their related function in mediating development of follicles. The primary (PF) to secondary follicle (SF) transition is a key link in the early stages of folliculogenesis^44^. In comparison to the normal ovary with many PFs and SFs in egg-laying hens, the atrophic ovaries of broody chickens had numerous PFs but few SFs, suggesting that they lacked the primary to secondary follicle transition. Consistent with this, in the AO there was lower expression of heat shock protein 90 (*HSP90*), *MMP2*, G protein subunit alpha 11 (*GNA11*), and gamma-aminobutyric acid type B receptor (*GABBR*) genes, which encode important enzymes or signaling factors in intracellular signaling of hormones regulating folliculogenesis^45,46^. We also detected other reproductive-associated genes including dopamine beta-hydroxylase (*DBH*), inhibin beta A/B (*INHBA/B*), and oxytocin receptor (*OXTR*). Compared with previous observations using suppression subtractive hybridization (SSH)^8^, we did not find *AMHRII*, *PRLR*, and *ERα* genes differentially expressed. The reason for this discrepancy is likely that tissues were collected at different broody stages. Qi *et al*.^47^ showed that SSH often presents a few false positives, whereas RNA-seq provides an unbiased methodology to investigate the gene expression pattern through deep sequencing.

Cell growth and death involve diverse complex cellular processes such as proliferation, differentiation, transformation, survival, and apoptosis, and changes in related genes and kinases^35–39^. Cytoplasmic Smad proteins transduce TGF-beta signals to result in granulosa cell proliferation, thereby leading to proper follicular development within ovarian tissues^35^. The mitogen activated protein kinase family (*MAPK*), consisting of the extracellular signal regulated protein kinases, have been implicated in various cellular processes involving cell growth, proliferation, and development^36^. *GADD45* is necessary for G_2_/M checkpoint control in the cell cycle, and is involved in DNA replication, cell proliferation, and survival^37^. Increased expression of *GADD45* causes cell cycle arrest following DNA damage^37^. S-phase kinase-associated protein 2 (*SKP2*), which is a member of the F-box protein family, mediates cell proliferation and cell cycle regulation by degrading cyclins^38^. Receptor-interacting protein kinase (*RIPK*)-1 is a master regulator of cell survival inhibiting *RIPK3*-mediated necroptosis^39^. In the current study, strongly suppressed expression of *SMAD2*, *MAPK11*, *SKP2*, *RIPL1*, and *GADD45* overexpression, along with phenotypic results collectively suggest that the broody chickens have relatively weaker cell growth and development in the ovary.

Compared to the abundant ovaries in egg-laying hens, the absence of yellow follicles in broody chickens was closely related to the reduced expression of genes significantly enriched in many signal transduction pathways including PI3K-Akt signaling, cGMP-PKG signaling, TGF-beta signaling, and Hippo signaling pathways, resulting in relatively weaker tissue development. Krishna *et al*. found that the PI3K-Akt pathway played an important role in the oocyte for resumption of meiosis and maturation of mouse oocytes and involved the Pten gene^48^. Cheng *et al*. observed that activation of the NO-cGMP-PKG pathway stimulated osteoblast differentiation and maturation in rats under a sinusoidal electromagnetic field, indicating that the cGMP-PKG pathway has significant functions in mediating cell differentiation and maturation^49^. According to multiple studies, the TGF-beta pathway promotes granulosa cell proliferation and follicular growth in the antral follicle stage^31,35,47^. A recent study showed, interestingly, that Hippo is a critical signaling pathway in regulating tissue regeneration, organ size, and stem cell self-renewal via suppressing cell growth^50^. In our study, we observed reduced expression of genes related to the above-mentioned pathways in AO, indicating that broodiness in chicken affected proliferation of granulosa cells and oocyte maturation mediated by the above pathways. In addition, the target genes of miRNAs and lncRNAs were also found to be enriched in the four pathways.

Expression of pro-apoptotic factors such as caspase 6 (*CASP6*) and caspase 7 (*CASP7*) was decreased. *CASP6/7* are aspartate-specific cysteine proteases, members of the caspase family associated with apoptosis, and have critical functions in apoptosis execution^51^. We also found that the level of calpain 2 (*CAPN2*), known to activate caspase 12 which participates in endoplasmic reticulum stress-induced apoptosis and to convert Bcl-xL to a pro-apoptotic molecule from an anti-apoptotic one, was reduced^51^. These results suggest that broodiness was associated with reduced apoptosis via the HPG axis on approximately the 30^th^ day of the broody period. However, Jing *et al*. suggested that goose broodiness involves decreased granulosa cell proliferation and increased apoptosis in the preliminary stage of the broody period^52^. Altogether, the above results suggest that the avian HPG axis has a stage-specific mediation of ovary development or tissue morphology during the broody period. For example, reduced cell proliferation and apoptosis can occur at the same time to maintain tissue homeostasis because of the highly atrophic status of broody chicken ovaries.

Several groups have investigated miRNA expression in tissues of broody birds. Chen *et al*. identified 94 and 114 novel miRNAs in the hypothalami of the egg-laying and broody goose, respectively, and detected 52 DEMs, using the Solexa sequencer^53^. Yu *et al*. showed that conjoint comparisons in three kinds of preovulatory follicles in broody and egg-laying geese revealed 44 DEMs using Illumina Hiseq. 2500^52^. Our findings corroborate the previous in that miRNAs with the highest expression in the chicken ovary were also highly abundant in the goose ovary^15^. The high conservation of miRNA between species implies that these miRNAs have critical biological functions. We detected 116 DEMs, many of which are related to ovarian function. For example, miR-34b and miR-34c, two well-known miRNAs, are targets of p53 and cooperate in suppressing adhesion-independent growth and proliferation in mouse ovarian surface epithelial cells^54^. MiR-18, miR-98, miR-128, miR-135, and miR-148 affect ovarian cell steroidogenesis, including the production of progesterone, testosterone, and estradiol^17^. Recently, the miR-200 family was implicated in ovarian cancer initiation and progression via stage-specific regulation^55^. Moreover, we revealed the potential function of a few miRNAs in the chicken ovary through pathway analysis of intersection genes. Our results provide novel information regarding the regulatory roles of these miRNAs.

In recent years, lncRNA have received increased attention for their involvement in various aspects of tissue development (for example, bursa of fabricius and muscle)^24,25^. This experiment is the first to show expression profiles of lncRNA transcripts in the chicken ovary and DE lncRNA transcripts in response to broodiness using whole transcriptome sequencing. Through functional analysis, 1,001 target genes of DE lncRNA transcripts were enriched in 6,267 Go terms and 270 pathways, the results of which demonstrate that lncRNAs have important roles in ovarian function. Through interaction analysis of protein-coding transcripts, lncRNA transcripts, and miRNAs, we also discovered that lncRNA transcripts could compete for miRNAs binding sites with protein-coding genes and subsequently influence their expression. The genes associated with cell proliferation, such as *GADD45* and *SMAD2*^35,37^, and those related to reproductive processes including *CYP1B1* and *MMP2*^43,45^, could be modulated by several lncRNAs. Moreover, *CASP6*, a key factor in apoptosis execution^51^, has a relatively lower expression in the AO of BC and was found to be under the control of the highly expressed lncRNAs. This provides direct evidence that these lncRNAs, as regulators of gene expression, could modulate multiple subsystems involved in ovarian development in response to broodiness.

Integration of multi-omics can generate new knowledge that is not accessible by analysis of single datasets alone^56,57^. For example, Robertson *et al*. demonstrated that the integration of three datasets improved classification accuracy to ~89% from the average of individual datasets at ~68.5%^58^. Justin *et al*. integrated diverse genomic, transcriptomic, and phosphoproteomic datasets to identify altered processes in the phosphorylation status of prostate cancer cells, and provided a reliable reference for drug prioritization^59^. In this study, we improved the validity of functional analysis of DE miRNAs using intersection genes. In addition, we constructed a ceRNA relationship network by integrating multiple omics analyses, and subsequently detected miRNA and lncRNA highly relevant to ovary development in broody chickens.

Because of high sensibility, RNA-seq is also a powerful tool to determine transcriptional structure and alternative splicing of genes^31,60^. In our study, we detected numerous novel protein-coding transcripts and 79.63% protein-coding genes that had novel transcripts, suggesting that the expression of these protein-coding genes has obvious tissue-specificity in chicken ovary. Due to its widespread usage and molecular versatility, alternative splicing emerges as a central element in gene regulation that increases the diversity of genes expression and function^61^. Previous studies evidenced that changes individual alternative splicing isoform are small, but numerous splicing programs resulted in strong effects on cell function^61–64^. Our study detected a large number of alternative splicing events including TSS, TTS and SKIP, implying that vast novel protein-coding transcripts identified were mainly generated by these alternative splicing events, which play critical roles in the development and maintenance of laying hens ovaries.

## Conclusion

In summary, we characterized mRNA, miRNA, and lncRNA transcript profiles of the chicken ovary by RNA-seq. We identified and characterized three kinds of DE genes that were involved in ovary physiology in broody chickens. We also constructed regulatory networks of the molecular mechanisms of ovarian atrophy. This study expanded our understanding of the molecular mechanisms underlying reproductive system development and maintenance in laying hens. Such information may also have relevance to understanding reproductive disorders in humans.

## Materials and Methods

### Ethics statement

All animal care and experimental procedures were approved by the Institutional Animal Care and Use Committee of Sichuan Agricultural University (No. YYS130125). All research work was conducted in strict accordance with the Sichuan Agricultural University (SAU) Laboratory Animal Welfare and Ethics guidelines.

### Animals

Total 400 laying Dongxiang blue-shelled (a native breed exhibiting high broodiness) hens were reared at the poultry farm of Sichuan Agricultural University (Sichuan, China). At 380 days of age, birds were selected randomly and divided into two groups: egg-laying and broody. All selected birds had identical genetic background and appearance, and chickens in each group had a similar body weight. Egg-laying hens had a similar egg-laying pattern including the ovipository cycle and daily egg-laying time. Broody birds persistently nested and incubated for approximately 30 consecutive days, and their ovaries presented as atrophic.

### Morphology, hormones, and immunohistochemistry assays

Six chickens from each group were anesthetized with sodium pentobarbital and euthanized. Complete ovaries were collected and placed in dissecting pans, and morphological characteristics and weight data were recorded. The number of white follicles (WF, 1–5 mm in diameter and have not entered the hierarchy), small yellow follicles (SYF, 5–10 mm in diameter and have not entered the hierarchy), and large yellow follicles (LYF, preovulatory follicles, >10 mm in diameter and have entered the hierarchy) were counted^65,66^. Stroma with cortical follicles <1 mm in diameter were dissected out of the ovaries, fixed in 4% paraformaldehyde with phosphate buffer (pH 7.4), embedded in paraffin, sectioned, and mounted on slides for hematoxylin and eosin staining. Histological characteristics of the stroma were observed using Advanced Research Software (Nikon) and an Eclipse 80i microscope (Nikon). A volume of 2 mL of blood was collected using venipuncture from each bird. After centrifugation at 2000× rpm for 10 min, pure plasma was immediately isolated from the blood supernatant and stored at −20 °C. We measured the plasma concentration of prolactin (PRL), luteinizing hormone (LH), and follicle stimulating hormone (FSH) using chicken-specific ELISA Kits (Abcam Inc., Cambridge, UK), following the manufacturer’s protocols.

### RNA extraction, library construction, and sequencing

Total RNA was isolated from three normal ovaries (NO) of egg-laying hens (EH) and three atrophic ovaries (AO) of broody chickens (BC) using Trizol RNA extraction reagent (Invitrogen Corp., CA, USA), following the manufacturer’s protocol. The concentration and purity of total RNA were assessed with spectrophotometry at wavelengths of 260, 280, and 230 nm using the NanoVue Plus Spectrophotometer, and the integrity of total RNA was evaluated with 2% agarose gel electrophoresis. The cDNA libraries of RNA (mRNA/lncRNA) and small RNA were generated from 10 μg and 2 μg of total RNA, respectively^24^. rRNA was removed using an Epicentre Ribo-zero rRNA Kit (Epicentre, USA). Sequencing was performed using Illumina HiSeqTM 2500 by Gene Denovo Biotechnology Co. (Guangzhou, China).

### Analysis of protein-coding and lncRNA transcripts

The raw data were subjected to quality check using FastQC (v0.11.4) (http://www.bioinformatics.babraham.ac.uk/projects/fastqc/). To obtain high quality clean reads, we removed low quality reads containing more than 50% of low quality (Q-value ≤ 20) bases, reads containing more than 10% of unknown nucleotides, and reads containing adapters from raw reads. After the rRNA mapped reads were removed using Bowtie 2^67^, mapping reads to the rRNA database, the remaining reads were aligned with the chicken reference genome using TopHat2 (version 2.0.3.12)^68^. The reconstruction and identification of transcripts was carried out with the software Cufflinks^69^, TopHat2, Cuffmerge, and Cuffcompare. The programs Coding-Non-Coding-Index (CNCI) (version 2)^70^, Coding Potential Calculator (CPC)^71^ (http://cpc.cbi.pku.edu.cn/) and phylogenetic codon substitution frequency (PhyloCSF)^25^ were used to predict the protein-coding potential of new transcripts with default parameters. The intersection of the results without protein-coding potential yielded lncRNA transcripts. The target genes for lncRNA transcripts were predicted through cis- and trans-regulation analysis^24^. The expression level of all transcripts was normalized using FPKM (Fragments Per Kilobase of transcript per Million mapped reads) with the software RSEM^72^. Transcripts with a false discovery rate (FDR) <0.05 and fold change ≥2 were then identified as significant differentially expressed protein-coding or lncRNA transcripts using edgeR package (http://www.r-project.org/). The details were supplied in additional file 3.

### miRNA analysis

To obtain clean reads, raw reads were further filtered according to the following rules: (1) Removing low quality reads containing more than one low quality (Q-value ≤ 20) base or containing unknown nucleotides (N); (2) Removing reads without 3′ adapters; (3) Removing reads containing 5′ adapters; (4) Removing reads containing 3′ and 5′ adapters but no small RNA fragment between them; (5) Removing reads containing polyA in the small RNA fragment; and (6) Removing reads shorter than 18 nt (not including adapters). The clean reads were aligned with the GenBank database (Release 209.0), the Rfam database (11.0), and the reference genome to identify and remove rRNA, scRNA, snoRNA, snRNA, tRNA, repeat sequences, and fragments from mRNA degradation. The remaining reads were searched against miRBase 21.0 to identify known miRNAs in chicken and known miRNAs in other species. Based on their genome position and hairpin structures predicted by the software Mireap_v0.2, novel miRNA candidates were identified^73^. The miRNA expression level was calculated and normalized to transcripts per million (TPM). We identified miRNAs with a FDR < 0.05 and fold change ≥2 as significant differentially expressed. TargetScan (Version 7.0), Miranda (v3.3a), and RNAhybrid (v2.1.2) + svm_light (v6.01) were used to predict targets of miRNA. The details were supplied in additional file 3.

### Functional enrichment analysis

Gene Ontology (GO) analysis of the differentially expressed and target genes was performed with the software DAVID^25^. KEGG (Kyoto Encyclopedia of Genes and Genomes) pathway analysis for differentially expressed and target genes was carried out with the software KOBAS v2.0 using a hypergeometric test^24^. The results with P-value < 0.05 were considered to be significantly enriched.

### Interaction analysis for protein-coding, miRNA, and lncRNA transcripts

Based on the miRNA-lncRNA transcript and miRNA- protein-coding transcript relationships and competitive combination with miRNAs, we constructed an lncRNA transcript -miRNA- protein-coding transcript ceRNA network. The ceRNA theory is applied to investigate the functions of lncRNA^34^. A lncRNA transcript can bind a given miRNA and thereby derepress the target protein-coding transcript^24^. Cytoscape 3.4.0 was used to analyze and visualize the interaction analysis.

### Validation by real time quantitative PCR (RT-qPCR)

Five protein-coding genes and lncRNA transcripts were selected to validate RNA-seq results using RT-qPCR. First-strand cDNA was synthesized from 500 ng total RNA with the PrimeScript RT reagent Kit (Perfect Real-Time; TaKaRa, Dalian, China) following recommendations of the manufacturer. The β-actin gene was used as the endogenous control for normalization and all primers are shown in Table S1. RT-qPCR assays were also performed to determine miRNA expression, and the U6 snRNA served as a housekeeping gene. Briefly, after miRNA were separated from ovaries using the mirVana miRNA isolation kit (Abcam Inc., Cambridge, UK), 3 μg of miRNA were subjected to reverse transcription with the One Step PrimeScript® miRNA cDNA Synthesis Kit (Tiangen, China). All reactions were carried out in the CFX96 qPCR system (Bio-Rad, USA) with triplicate reactions for each sample^74^. The quantification of relative expression of protein-coding genes, lncRNA transcripts, and miRNAs was performed using the 2^−ΔΔCt^ method. The Pearson’s correlation between RNA-seq and RT-PCR results was determined with SAS 9.3 (SAS Inst., Cary, North Carolina, USA).

### Statistical analysis

Results are expressed as means ± standard deviation of the mean. Data were subjected to one-way analysis of variance (ANOVA) with Duncan’s Multiple Range test used for pairwise comparisons, using SAS 9.3 (SAS Inst., Cary, North Carolina, USA). Values were considered to be significantly different at P < 0.05.

### Availability of data and materials

The raw data has been submitted to the National Center for Biotechnology Information (NCBI) Sequence Read Archive (SRA), and the accession number is PRJNA412674.

## Electronic supplementary material

Supplemental tables and figures

Table S7 and S8

Supplemental materials and methods
