# Supplementary material for: Whole-transcriptome analysis of atrophic ovaries in broody chickens reveals regulatory pathways associated with proliferation and apoptosis
Source: Sci Rep. 2018 May 8;8:7231. doi: 10.1038/s41598-018-25103-6 (PMC5940789; doi:10.1038/s41598-018-25103-6)
Supplement: Supplementary file 1 — Supplemental tables and figures [file 41598_2018_25103_MOESM1_ESM.pdf]

Whole-transcriptome analysis of atrophic ovaries in broody chickens reveals regulatory pathways associated with proliferation and apoptosis

Lingbin Liu[1,2], Qihai Xiao[1], Elizabeth R. Gilbert[2], Zhifu Cui[1], Xiaoling Zhao[1], Yan Wang[1], Huadong Yin[1], Diyan Li[1], Haihan Zhang[2], Qing Zhu[1]

[1]Farm Animal Genetic Resources Exploration and Innovation Key Laboratory of Sichuan Province, Sichuan Agricultural University, Chengdu Campus, 611130, Sichuan Province, China.

[2]Department of Animal and Poultry Sciences, Virginia Tech, Blacksburg, 24061, Virginia, USA.

Lingbin Liu and Qihai Xiao contributed equally to this work.

Correspondence Author: Qing Zhu, Institute of Animal Genetics and Breeding, Farm Animal Genetic Resources Exploration and Innovation Key Laboratory of Sichuan Province, Sichuan Agricultural University, Ya'an, 625014, P.R.China.

E-mail: zhuqingsicau@163.com;

Telephone number: +86-835-2882006;

Additional file 1. Supplemental tables and figures

Table S1. Primers used for real time PCR

Genes	Gene name[1]	Primers(5'-3')	Length
Protein-coding gene	*CASP6*	F: TGACCCTGCAGAGCAATACA	153
		R: CCAAGGTCTGTCAAACTGCG	
	CYP1B1	F: CATCTTCCTCATCAGGTATCCAAAAGT	130
		R: GTACAGGAAAGCCACGATGTAG	
	GADD45	F: CCCACTGATCTCCATTGCGT	105
		R: CATATAGCGACTCTCCCGGC	
	MMP2	F: CGATGCTGTCTACGAGTCCC	96
		R: TAGCCCCTATCCAGGTTGCT	
	SMAD2	F: GAGAGGTTGGTGTGCTACGC	143
		R: ACAACCGGTGGAGTGAATGG	
	β-actin	F: GAGAAATTGTGCGTGACATCA	152
		R: CCTGAACCTCTCATTGCCA	
lncRNA	XLOC_005141	F: ATGGCTTCTGCTGGATTGCT	159
		R: GGAACCAGAGCCAGACCTTC	
	XLOC_008879	F: CCAACAGCCTAAGAACCA	151
		R: TTAGAGCCACAGTCCCATT	
	XLOC_016063	F: AGTACAATAGGCATCTCCATC	261
		R: TCCCTTCCAGGTCTACATT	
	XLOC_021592	F: GGAAATGCCACTGGAGGTCA	197
		R: CCATAGGCCATTTGGCTTGC	
	XLOC_027660	F: AACCCTCTGCCTTCGTTCTG	92
		R: CTGAATGCCCACCTGTCAGT	

[1] *CASP6*: caspase 6; *CYP1B1*: cytochrome P450 family 1 subfamily B member 1; *GADD45*: Growth arrest and DNA damage-inducible 45; *MMP2*: matrix metallopeptidase 2; *SMAD2*: SMAD family member 2.

Table S2. Compare of ovarian morphological characteristics between egg-laying and broody chickens

Traits	EH	BC
Body weight (kg)	2.47 ±0.11*	1.65 ±0.13
Ovary weight (g)	48.17 ±4.43**	2.67 ±0.06
Ovary ratio (%)	1.95 ±0.18**	0.16 ±0.02
LYF (>10mm, count)	5.33 ±1.15	\
SYF (5~10mm, count)	11.33 ±0.58	\
WF (1~5mm, count)	18.33 ±4.93	18 ±2.65
Stroma weight (g)	6.9 ±1.13**	2.67 ±0.06

Ovary ratio = Ovary weight / Body weight *100%. EH means egg-laying hen, and BC means broody chicken. LYF means the number of large yellow follicle, SYF means the number of small yellow follicle, and WF means the number of white follicle, stroma weight means the weight of stroma with cortical follicles < 5 mm in diameter. Results are expressed as mean ± standard deviation (n = 6). *, $P < 0.05$; **, $P < 0.0001$.

Table S3. Filtering information of raw reads[1]

Sample	Raw Reads Num	Clean Reads Num(%)	adapter(%)	low quality(%)	N[2](%)
NO1	80,966,554	79,949,512 (98.74%)	490,709 (1.21%)	17,812 (0.04%)	0 (0%)
NO2	91,626,870	90,447,086 (98.71%)	571,037 (1.25%)	18,855 (0.04%)	0 (0%)
NO3	83,822,790	82,772,486 (98.75%)	506,737 (1.21%)	18,415 (0.04%)	0 (0%)
AO1	86,866,082	85,840,896 (98.82%)	484,632 (1.11%)	27,961 (0.06%)	0 (0%)
AO2	84,523,568	83,475,304 (98.76%)	507,390 (1.2%)	16,742 (0.04%)	0 (0%)
AO3	81,020,796	79,931,822 (98.66%)	526,499 (1.3%)	17,988 (0.04%)	0 (0%)

[1]NO represents normal ovary of egg-laying hen; AO denotes atrophic ovary of broody chicken.
[2]unknown nucleotide.

Table S4. Characteristics of the reads from RNA sequencing libraries in chicken ovary[1]

Sample[1]	Before Filter				After Filter			
	Raw Data[1] (bp)	Q20 (%)	N (%)	GC (%)	Clean Data[2] (bp)	Q20 (%)	N[2] (%)	GC (%)
NO-1	10,120,819,250	9,872,382,417 (97.55%)	55,768 (0.00%)	5,429,787,187 (53.65%)	9,993,689,000	9,750,901,388 (97.57%)	55,079 (0.00%)	5,363,131,635 (53.67%)
NO-2	11,453,358,750	11,180,124,656 (97.61%)	65,818 (0.00%)	5,933,819,015 (51.81%)	11,305,885,750	11,038,940,291 (97.64%)	64,960 (0.00%)	5,857,839,762 (51.81%)
NO-3	10,477,848,750	10,215,045,171 (97.49%)	59,906 (0.00%)	5,501,749,284 (52.51%)	10,346,560,750	10,089,737,147 (97.52%)	59,146 (0.00%)	5,433,879,090 (52.52%)
AO-1	10,858,260,250	10,526,669,054 (96.95%)	49,225 (0.00%)	5,928,942,893 (54.60%)	10,730,112,000	10,406,573,851 (96.98%)	48,599 (0.00%)	5,859,100,999 (54.60%)
AO-2	10,565,446,000	10,325,532,705 (97.73%)	60,257 (0.00%)	5,770,700,285 (54.62%)	10,434,413,000	10,199,874,248 (97.75%)	59,464 (0.00%)	5,700,360,944 (54.63%)
AO-3	10,127,599,500	9,874,744,924 (97.50%)	57,585 (0.00%)	5,359,942,164 (52.92%)	9,991,477,750	9,744,783,499 (97.53%)	56,800 (0.00%)	5,288,577,814 (52.93%)

[1]NO represents normal ovary of egg-laying hen; AO denotes atrophic ovary of broody chicken.
[2]unknown nucleotide.

Table S5. Alignment information of clean reads with ribosome RNA (rRNA) database

Sample	Clean Reads Num	Mapped Reads	Unmapped Reads
NO1	79,949,512	55,100 (0.07%)	79,894,412 (99.93%)
NO2	90,447,086	71,690 (0.08%)	90,375,396 (99.92%)
NO3	82,772,486	54,112 (0.07%)	82,718,374 (99.93%)
AO1	85,840,896	45,222 (0.05%)	85,795,674 (99.95%)
AO2	83,475,304	55,386 (0.07%)	83,419,918 (99.93%)
AO3	79,931,822	35,110 (0.04%)	79,896,712 (99.96%)

NO represents normal ovary of egg-laying hen; AO denotes atrophic ovary of broody chicken.

Table S6. Alignment information of clean reads with reference genome

Sample	Clean Reads	Unmapped Reads	Unique Mapped Reads	Multiple Mapped reads
NO1	79,894,412	8,253,504(10.33%)	71,165,130(89.07%)	475,778(0.6%)
NO2	90,375,396	9,745,039(10.78%)	80,151,261(88.69%)	479,096(0.53%)
NO3	82,718,374	9,329,750(11.28%)	72,956,480(88.2%)	432,144(0.52%)
AO1	85,795,674	10,872,578(12.67%)	74,367,092(86.68%)	556,004(0.65%)
AO2	83,419,918	9,946,842(11.92%)	72,895,508(87.38%)	577,568(0.69%)
AO3	79,896,712	8,759,894(10.96%)	70,687,346(88.47%)	449,472(0.56%)

NO represents normal ovary of egg-laying hen; AO denotes atrophic ovary of broody chicken.

Table S7. Gene ontology enrichment analysis of differentially expressed protein-coding transcripts (DEGs).

Table S8. KEGG pathway analysis of differentially expressed protein-coding transcripts (DEGs).

Table S9. Candidate genes involved in reproductive endocrine system, and cell growth and death pathways.

Symbol	log2(FC[1])	P-value	FDR[2]	Description
Reproductive endocrine system				
DBH	7.63	6.28E-07	5.65E-05	dopamine beta-hydroxylase
TH	4.15	2.43E-04	6.36E-03	tyrosine hydroxylase
OXTR	2.47	5.53E-06	3.39E-04	oxytocin receptor
PRKAB2	1.12	2.82E-05	1.26E-03	protein kinase AMP-activated non-catalytic subunit beta 2
HSP90	-1.11	1.38E-03	2.12E-02	heat shock protein 90
CYP1B1	-1.17	2.14E-04	5.76E-03	cytochrome P450 family 1 subfamily B member 1
MMP2	-1.20	5.19E-06	3.22E-04	matrix metallopeptidase 2
GUCY1A2	-1.21	1.76E-05	8.67E-04	guanylate cyclase 1 soluble subunit alpha 2
MYL9	-1.32	1.89E-07	2.07E-05	myosin light chain 9
ROCK2	-1.46	4.83E-03	4.94E-02	Rho associated coiled-coil containing protein kinase 2
ROCK1	-1.53	1.91E-03	2.63E-02	Rho associated coiled-coil containing protein kinase 1
GNA11	-1.59	6.93E-05	2.55E-03	G protein subunit alpha 11
GABBR	-2.10	9.23E-04	1.60E-02	gamma-aminobutyric acid type B receptor
MYLK	-2.25	4.41E-11	1.31E-08	myosin light chain kinase
HSD3B2	-2.30	5.02E-06	3.13E-04	hydroxy-delta-5-steroid dehydrogenase, 3 beta- and steroid delta-isomerase 2
INHBB	-3.10	1.09E-09	2.45E-07	inhibin beta B subunit
INHBA	-4.96	1.33E-12	4.97E-10	inhibin beta A subunit
Cell growth and death				
WEE2	1.35	4.50E-07	4.25E-05	WEE1 homolog 2
CDC20	1.20	1.39E-05	7.25E-04	cell division cycle 20
GADD45	1.13	1.14E-04	3.65E-03	Growth arrest and DNA damage-inducible 45
ORC2	1.11	2.90E-04	7.23E-03	origin recognition complex subunit 2
CDC25A	1.09	1.66E-05	8.29E-04	cell division cycle 25A
TP73	1.02	6.40E-04	1.24E-02	tumor protein p73
MAPK11	-1.03	8.62E-04	1.52E-02	mitogen-activated protein kinase 11
RIPK1	-1.13	1.12E-03	1.83E-02	receptor interacting serine/threonine kinase 1
CASP7	-1.21	1.10E-03	1.81E-02	caspase 7
CAPN2	-1.51	4.25E-06	2.73E-04	calpain 2
CASP6	-1.53	7.96E-05	2.82E-03	caspase 6
SKP2	-1.58	2.32E-08	3.46E-06	S-phase kinase-associated protein 2
SMAD2	-2.95	1.16E-25	1.53E-22	SMAD family member 2

[1]FC means fold change. Relative mRNA abundance of the gene when comparing the performance in the

broodiness ovary library to that in the egg-laying ovary library sequenced by deep sequencing.
[2]FDR represents false discovery rate.

Table S10. Characteristics of the reads (bp) from small RNA sequencing libraries in chicken ovary

Sample	Raw reads	High quality	3'adapter null	Insert null[2]	5'adapter	Smaller than 18nt	Poly A	Clean reads
NO1	13,000,645	12,700,883(97.69%)	17,973(0.14%)	37,231(0.29%)	6,972(0.05%)	171,134(1.35%)	249(0%)	12,467,324(98.16%)
NO2	13,332,221	12,996,485(97.48%)	21,575(0.17%)	110,266(0.85%)	12,772(0.1%)	248,975(1.92%)	544(0%)	12,602,353(96.97%)
NO3	13,821,253	13,482,018(97.54%)	22,125(0.16%)	86,291(0.64%)	10,380(0.08%)	208,384(1.55%)	197(0%)	13,154,641(97.57%)
AO1	12,783,658	12,486,606(97.67%)	16,781(0.13%)	104,464(0.84%)	7,310(0.06%)	118,962(0.95%)	201(0%)	12,238,888(98.01%)
AO2	12,932,457	12,610,547(97.51%)	23,225(0.18%)	34,719(0.28%)	10,365(0.08%)	193,950(1.54%)	400(0%)	12,347,888(97.92%)
AO3	12,450,148	12,161,532(97.68%)	18,837(0.15%)	59,283(0.49%)	6,539(0.05%)	105,107(0.86%)	184(0%)	11,971,582(98.44%)

[1]NO represents normal ovary of egg-laying hen; AO denotes atrophic ovary of broody chicken.
[2]Insert null means read containing 3’ and 5’ adapters but no small RNA fragment between them.

Supplemental Figure legends:

Figure S1. Total transcripts (A), and miRNAs (B) expression profile. NO represents normal ovary in egg-laying chicken; AO denotes atrophic ovary in broody chicken.

Figure S2. The statistics of novel transcripts coverage in distinct libraries.

Figure S3. The Ven diagram of transcripts identified in two distinct ovaries. (A) protein-coding transcripts; (B) lncRNA transcripts; (C) miRNAs.

Figure S4. The categories of alternative splicing events. AE: Alternative exon ends (5', 3', or both); XAE: Approximate AE (5', 3', or both); IR: Intron retention; XIR: Approximate IR; MIR: Multi-IR; XMIR: Approximate MIR; TSS: Alternative 5' first exon; TTS: Alternative 3' last exon; SKIP: Exon skipping; XSKIP: Approximate SKIP; MSKIP: Multi-exon SKIP; XMSKIP: Approximate MSKIP.

Figure S5. The category (A) and chromosome distribution (B) of all lncRNA transcripts.

Figure S6. GO/pathway analysis for intersection genes of DE miRNAs. (A) Top 20 significant changed GOs of intersection genes in biological process; blue and kermesinus show up-regulation and down-regulation in atrophic ovary of broody chicken, respectively. (B) Top 20 significant changed pathways of intersection genes.

Figure S7. GO/pathway analysis for target genes of DE lncRNA transcripts. (A) Top 20 significant changed GOs of target genes in biological process; blue and kermesinus show up-regulation and down-regulation in atrophic ovary of broody chicken, respectively. (B) Top 20 significant changed pathways of target genes.

Figure S8. The node degree distribution of ceRNA network.

Figure S9. Validation of RNA-seq data using real time quantitative PCR (RT-qPCR). The DE protein-coding genes (A), lncRNA transcripts (B), and miRNAs(C), were confirmed by RT-qPCR. Results are expressed as mean ± standard deviation; *, $P < 0.05$.

Figure S10. Correlation between RNA-seq and RT-qPCR results.

Figure S11. The expression patterns of four protein-coding gene-miRNA-lncRNA transcript pairs. The same color indicated the miRNA and its corresponding reciprocally expressed protein-coding gene and lncRNA transcript.

Figure S1

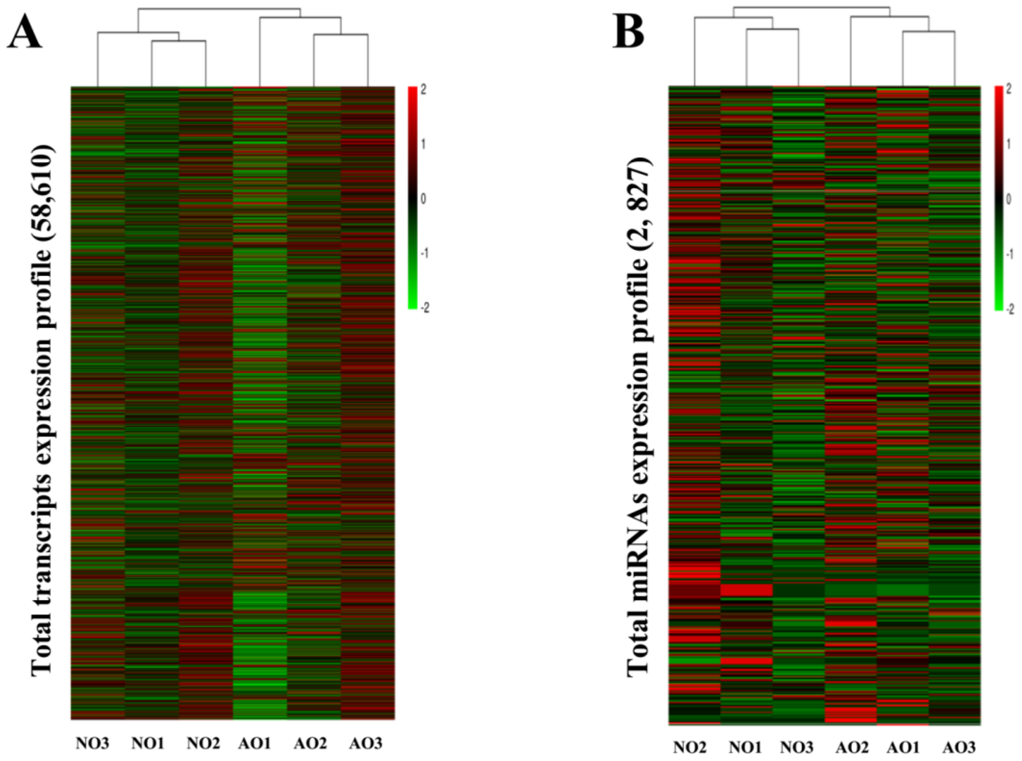
A
Total transcripts expression profile (58,610)
NO3
NO1
NO2
AO1
AO2
AO3
2
1
0
-1
-2
B
Total miRNAs expression profile (2, 827)
NO2
NO1
NO3
AO2
AO1
AO3
2
1
0
-1
-2

Figure S2

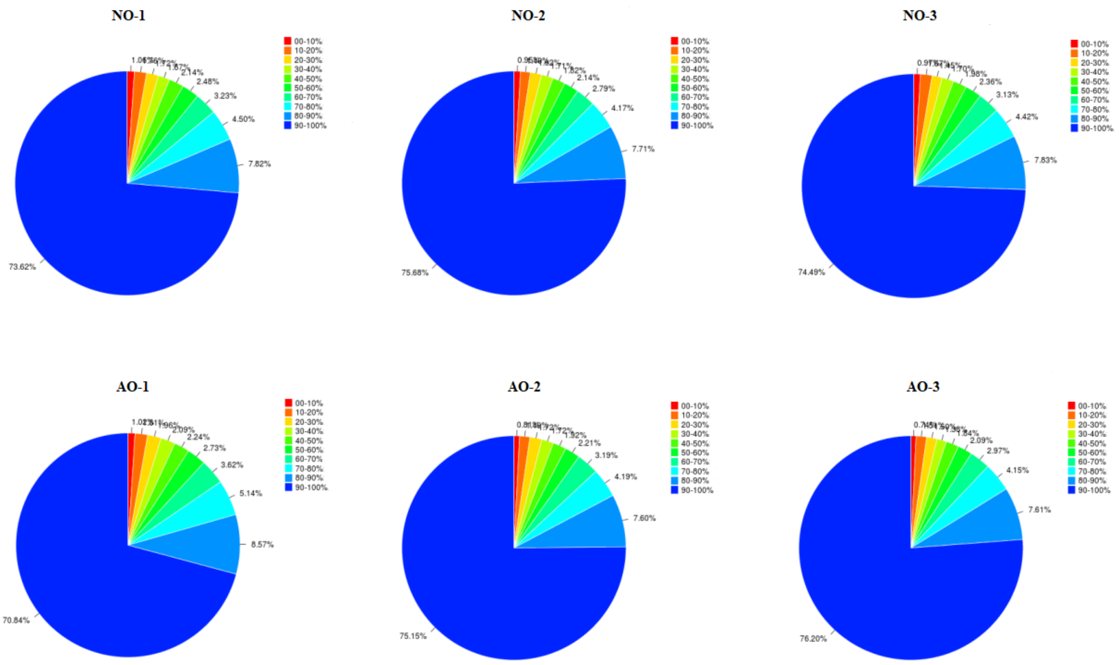
NO-1
NO-2
NO-3
AO-1
AO-2
AO-3
00-10%
10-20%
20-30%
30-40%
40-50%
50-60%
60-70%
70-80%
80-90%
90-100%
2.48%
3.23%
4.50%
7.82%
73.62%
2.79%
4.17%
7.71%
75.68%
2.36%
3.13%
4.42%
7.83%
74.49%
2.24%
2.73%
3.62%
5.14%
8.57%
70.84%
2.21%
3.19%
4.19%
7.60%
75.15%
2.09%
2.97%
4.15%
7.61%
76.20%

Figure S3

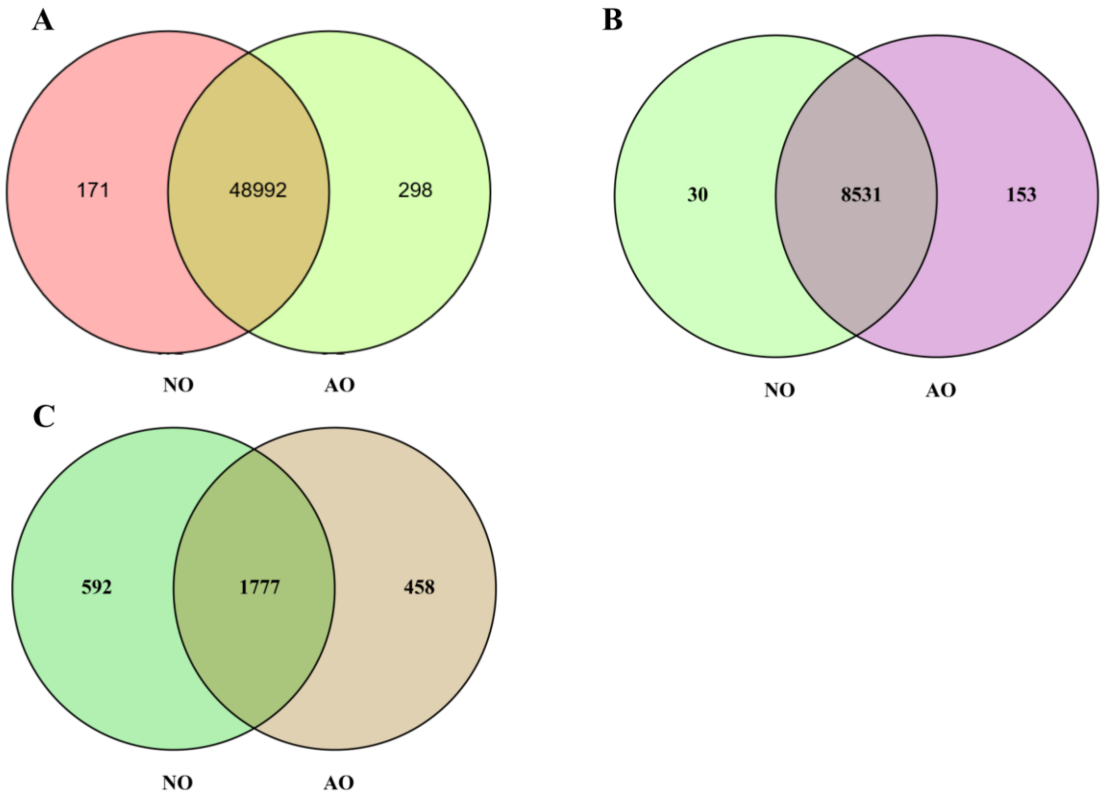

Figure S4

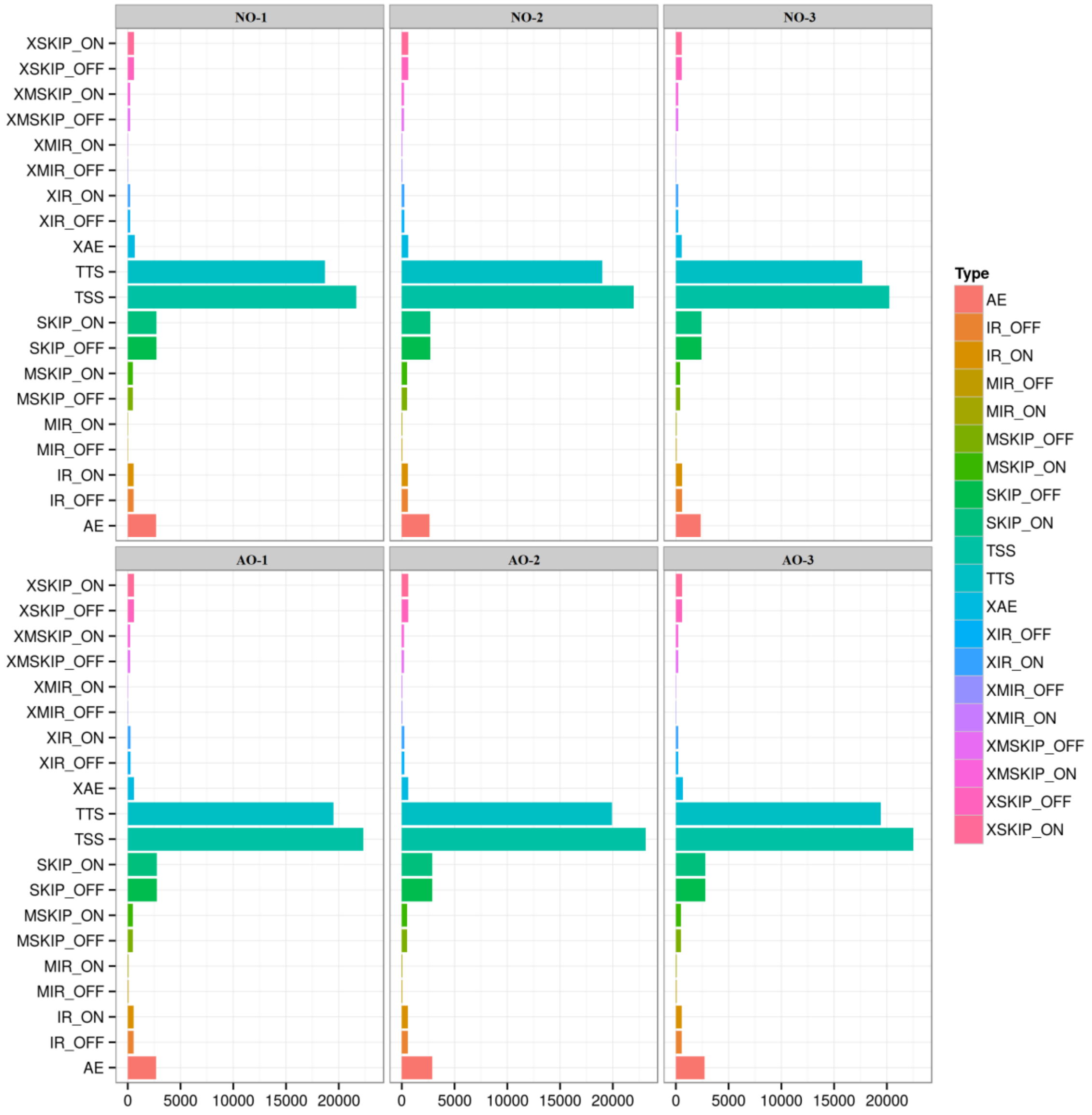
NO-1
NO-2
NO-3
AO-1
AO-2
AO-3
XSKIP_ON
XSKIP_OFF
XMSKIP_ON
XMSKIP_OFF
XMIR_ON
XMIR_OFF
XIR_ON
XIR_OFF
XAE
TTS
TSS
SKIP_ON
SKIP_OFF
MSKIP_ON
MSKIP_OFF
MIR_ON
MIR_OFF
IR_ON
IR_OFF
AE
0
5000
10000
15000
20000
Type
AE
IR_OFF
IR_ON
MIR_OFF
MIR_ON
MSKIP_OFF
MSKIP_ON
SKIP_OFF
SKIP_ON
TSS
TTS
XAE
XIR_OFF
XIR_ON
XMIR_OFF
XMIR_ON
XMSKIP_OFF
XMSKIP_ON
XSKIP_OFF
XSKIP_ON

Figure S5

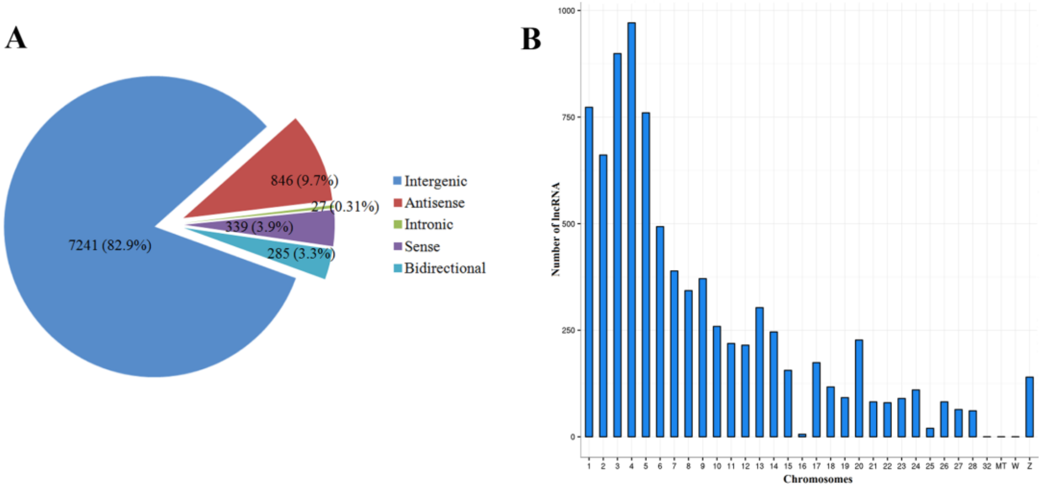
A
7241 (82.9%)
846 (9.7%)
27 (0.31%)
339 (3.9%)
285 (3.3%)
Intergenic
Antisense
Intronic
Sense
Bidirectional
B
Number of lncRNA
1000
750
500
250
0
1 2 3 4 5 6 7 8 9 10 11 12 13 14 15 16 17 18 19 20 21 22 23 24 25 26 27 28 32 MT W Z
Chromosomes

Figure S6

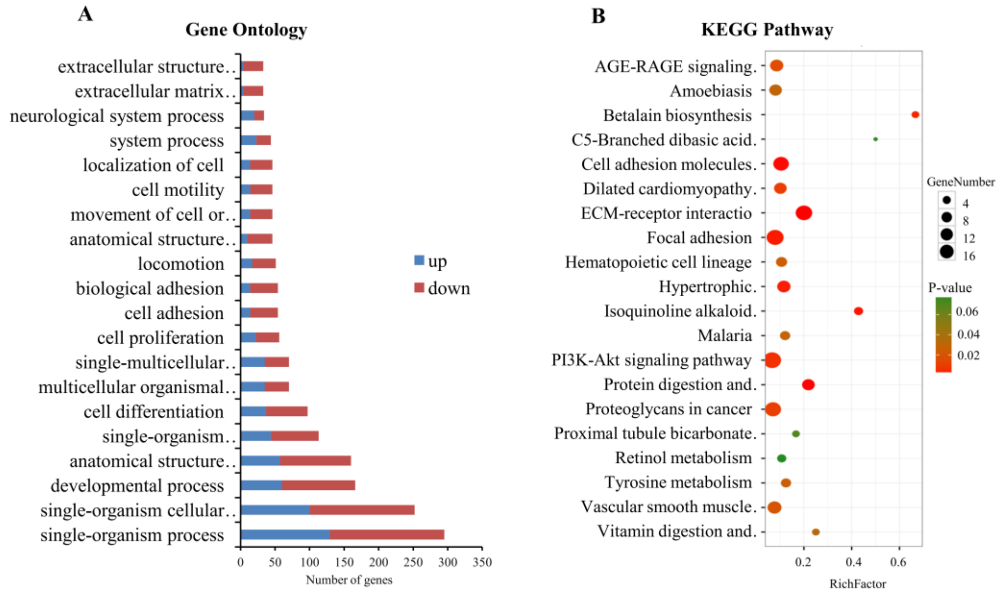
A
Gene Ontology
extracellular structure..
extracellular matrix..
neurological system process
system process
localization of cell
cell motility
movement of cell or..
anatomical structure..
locomotion
biological adhesion
cell adhesion
cell proliferation
single-multicellular..
multicellular organismal..
cell differentiation
single-organism..
anatomical structure..
developmental process
single-organism cellular..
single-organism process
up
down
0
50
100
150
200
250
300
350
Number of genes
B
KEGG Pathway
AGE-RAGE signaling.
Amoebiasis
Betalain biosynthesis
C5-Branched dibasic acid.
Cell adhesion molecules.
Dilated cardiomyopathy.
ECM-receptor interactio
Focal adhesion
Hematopoietic cell lineage
Hypertrophic.
Isoquinoline alkaloid.
Malaria
PI3K-Akt signaling pathway
Protein digestion and.
Proteoglycans in cancer
Proximal tubule bicarbonate.
Retinol metabolism
Tyrosine metabolism
Vascular smooth muscle.
Vitamin digestion and.
GeneNumber
4
8
12
16
P-value
0.06
0.04
0.02
0.2
0.4
0.6
RichFactor

Figure S7

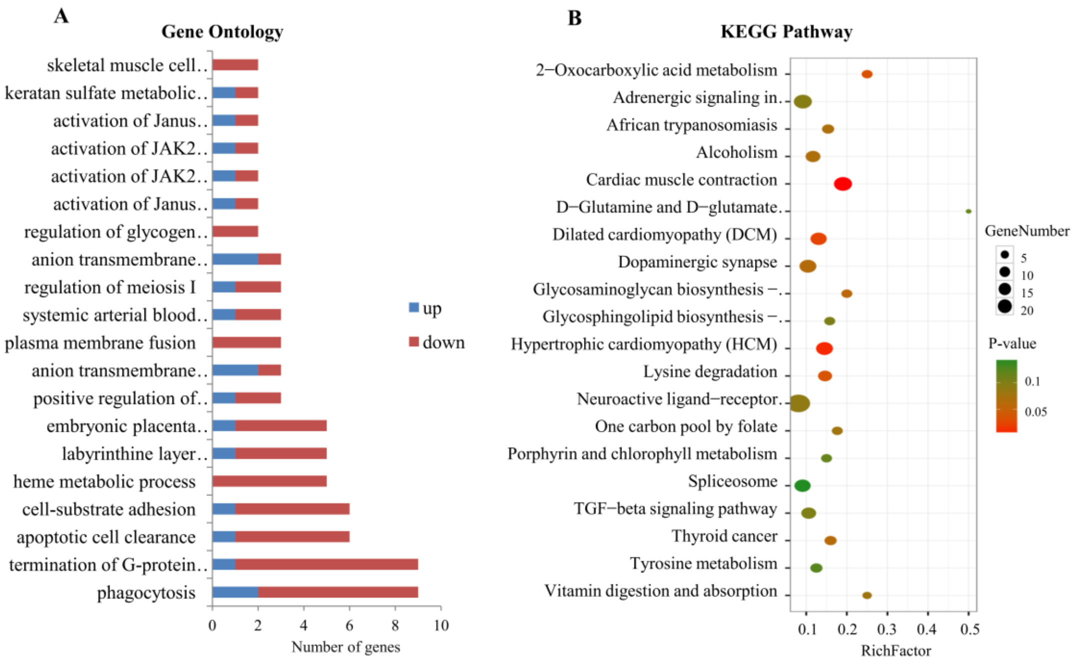

A
Gene Ontology
skeletal muscle cell..
keratan sulfate metabolic..
activation of Janus..
activation of JAK2..
activation of JAK2..
activation of Janus..
regulation of glycogen..
anion transmembrane..
regulation of meiosis I
systemic arterial blood..
plasma membrane fusion
anion transmembrane..
positive regulation of..
embryonic placenta..
labyrinthine layer..
heme metabolic process
cell-substrate adhesion
apoptotic cell clearance
termination of G-protein..
phagocytosis
0
2
4
6
8
10
Number of genes
up
down
B
KEGG Pathway
2−Oxocarboxylic acid metabolism
Adrenergic signaling in.
African trypanosomiasis
Alcoholism
Cardiac muscle contraction
D−Glutamine and D−glutamate.
Dilated cardiomyopathy (DCM)
Dopaminergic synapse
Glycosaminoglycan biosynthesis −.
Glycosphingolipid biosynthesis −.
Hypertrophic cardiomyopathy (HCM)
Lysine degradation
Neuroactive ligand−receptor.
One carbon pool by folate
Porphyrin and chlorophyll metabolism
Spliceosome
TGF−beta signaling pathway
Thyroid cancer
Tyrosine metabolism
Vitamin digestion and absorption
0.1
0.2
0.3
0.4
0.5
RichFactor
GeneNumber
5
10
15
20
P-value
0.1
0.05

Figure S8

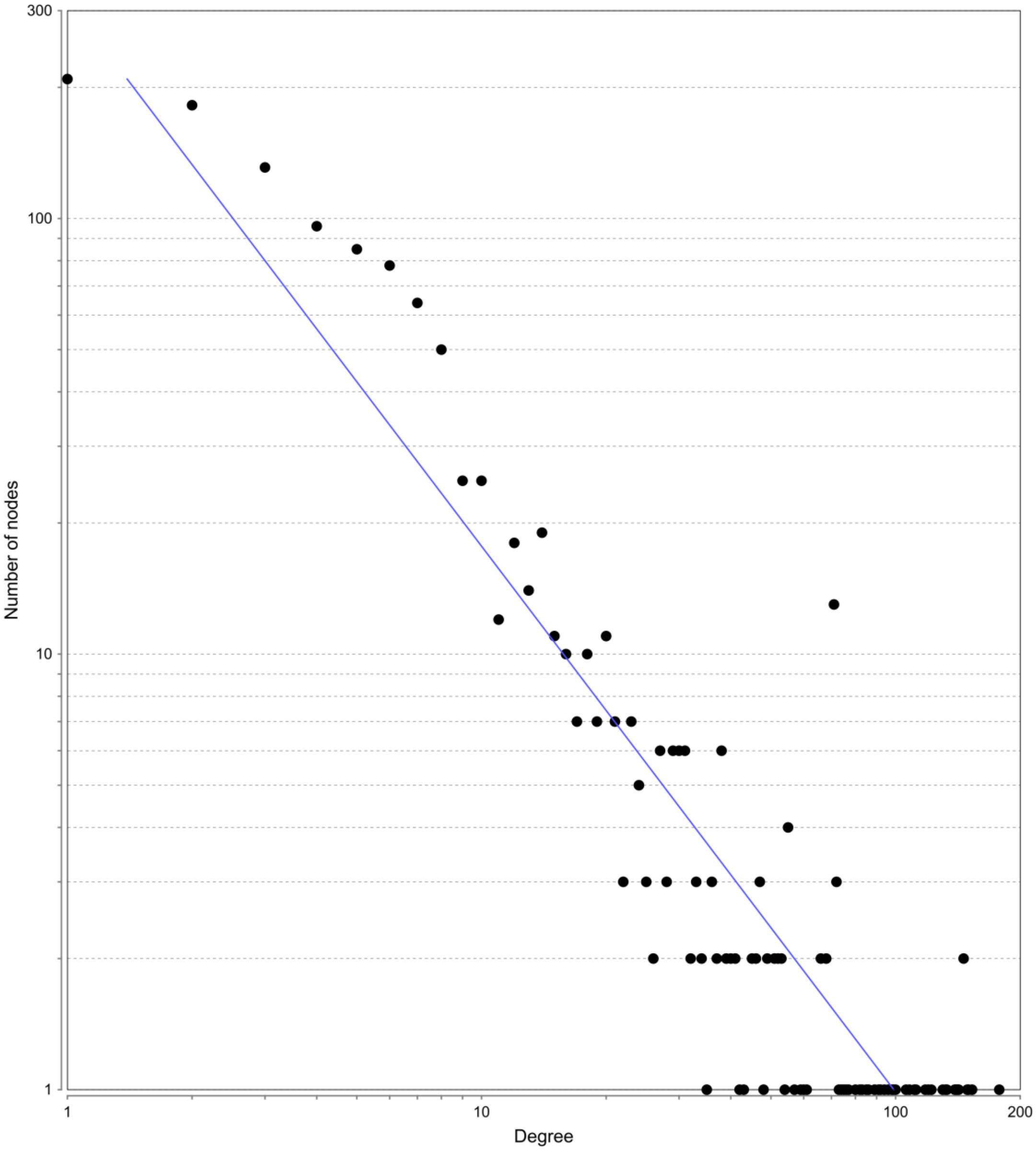

Figure S9

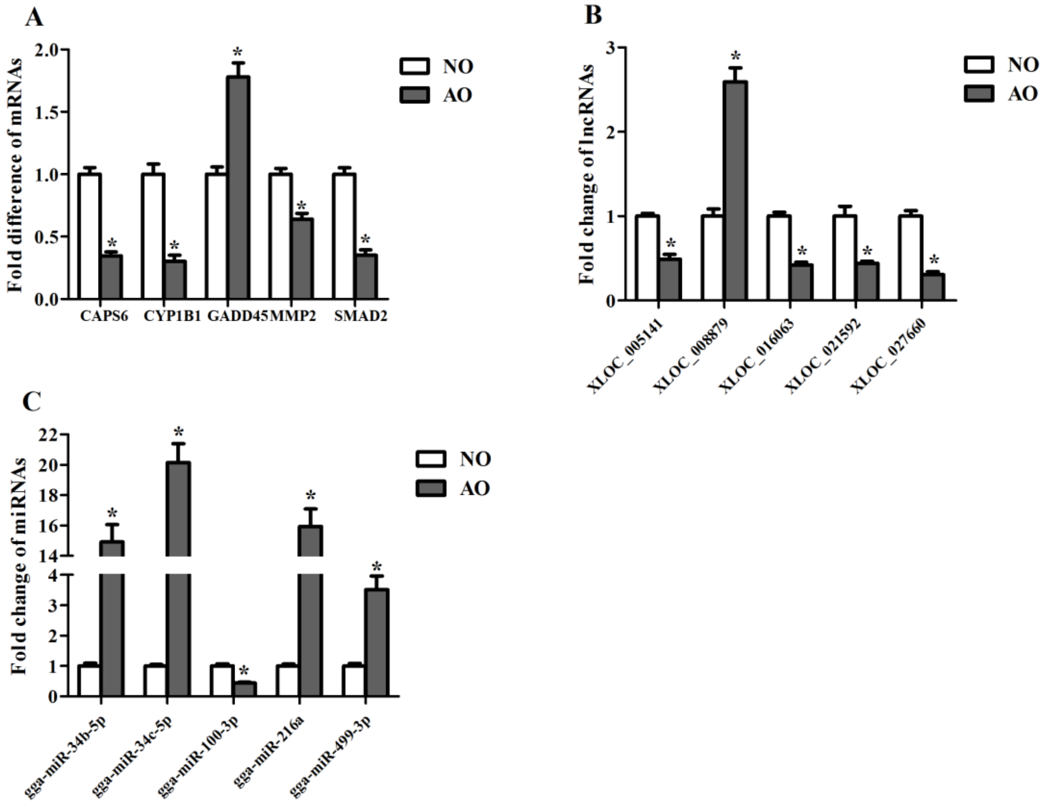
A
Fold difference of mRNAs
2.0
1.5
1.0
0.5
0.0
CAPS6
CYP1B1
GADD45
MMP2
SMAD2
NO
AO
B
Fold change of lncRNAs
3
2
1
0
XLOC_005141
XLOC_008879
XLOC_016063
XLOC_021592
XLOC_027660
NO
AO
C
Fold change of miRNAs
22
20
18
16
14
4
3
2
1
0
gga-miR-34b-5p
gga-miR-34c-5p
gga-miR-100-3p
gga-miR-216a
gga-miR-499-3p
NO
AO

Figure S10

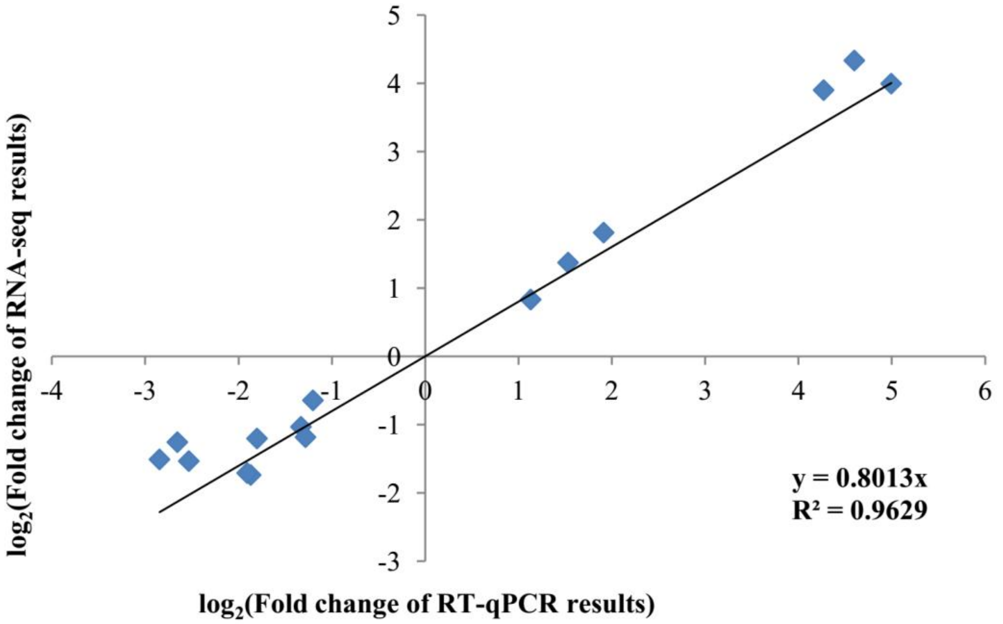

log2(Fold change of RNA-seq results)
log2(Fold change of RT-qPCR results)
y = 0.8013x
R² = 0.9629
5
4
3
2
1
0
-1
-2
-3
-4
-3
-2
-1
0
1
2
3
4
5
6

Figure S11

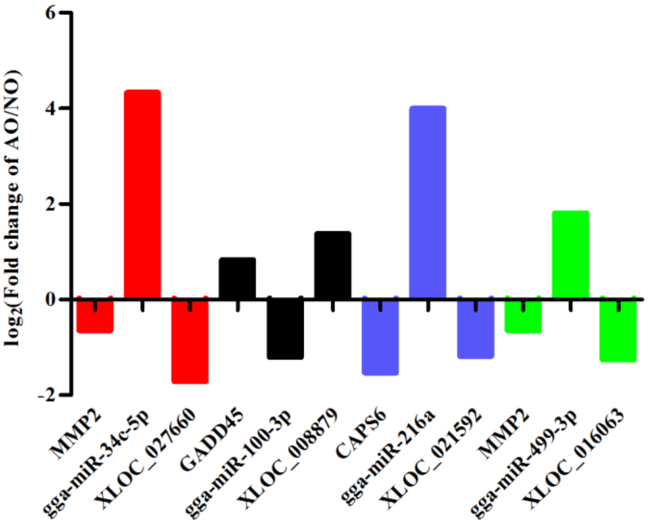

6
4
2
0
-2
log2(Fold change of AO/NO)
MMP2
gga-miR-34c-5p
XLOC_027660
GADD45
gga-miR-100-3p
XLOC_008879
CAPS6
gga-miR-216a
XLOC_021592
MMP2
gga-miR-499-3p
XLOC_016063
