# Supplementary material for: Whole-transcriptome analysis of atrophic ovaries in broody chickens reveals regulatory pathways associated with proliferation and apoptosis
Source: Sci Rep. 2018 May 8;8:7231. doi: 10.1038/s41598-018-25103-6 (PMC5940789; doi:10.1038/s41598-018-25103-6)
Supplement: Supplementary file 3 — Supplemental materials and methods [file 41598_2018_25103_MOESM3_ESM.pdf]

Whole-transcriptome analysis of atrophic ovaries in broody chickens reveals regulatory pathways associated with proliferation and apoptosis

Lingbin Liu[1,2], Qihai Xiao[1], Elizabeth R. Gilbert[2], Zhifu Cui[1], Xiaoling Zhao[1], Yan Wang[1], Huadong Yin[1], Diyan Li[1], Haihan Zhang[2], Qing Zhu[1]

[1]Farm Animal Genetic Resources Exploration and Innovation Key Laboratory of Sichuan Province, Sichuan Agricultural University, Chengdu Campus, 611130, Sichuan Province, China.

[2]Department of Animal and Poultry Sciences, Virginia Tech, Blacksburg, 24061, Virginia, USA.

Lingbin Liu and Qihai Xiao contributed equally to this work.

Correspondence Author: Qing Zhu, Institute of Animal Genetics and Breeding, Farm Animal Genetic Resources Exploration and Innovation Key Laboratory of Sichuan Province, Sichuan Agricultural University, Ya'an, 625014, P.R.China.

E-mail: zhuqingsicau@163.com;

Telephone number: +86-835-2882006;

Additional file 3. Supplemental metarials and methods

1. Analysis of protein-coding and lncRNA transcript

1.1 Filtering of Clean Reads

The raw data were subjected to quality check using FastQC[1] (v0.11.4) (http://www.bioinformatics.babraham.ac.uk/projects/fastqc/). Reads obtained from the sequencing machines included raw reads containing adapters or low quality bases which would affect the following assembly and analysis. Thus, to get high quality clean reads, reads were further filtered according to the following rules:

1) Removing reads containing adapters;

2) Removing reads containing more than 10% of unknown nucleotides (N);

3) Removing low quality reads containing more than 50% of low quality (Q-value≤20) bases.

1.2 Alignment with Ribosome RNA (rRNA)

To avoid the residual rRNA affecting the subsequent analysis, short reads alignment tool Bowtie2[2] was used for mapping reads to ribosome RNA (rRNA) database. The rRNA mapped reads were then removed; the maximum read mismatch is 5 bp. The remaining reads were further used in assembly and analysis of transcriptome.

1.3 Alignment with reference genome

The rRNA removed reads of each sample were then mapped to the chicken reference genome by TopHat2[3] (version 2.0.3.12), respectively. The alignment parameters were as follows:

1) Maximum read mismatch is 2;

2) The distance between mate-pair reads is 50 bp;

3) The error of distance between mate-pair reads is ±80 bp.

1.4 Transcripts Reconstruction

The reconstruction of transcripts was carried out with software Cufflinks[4] using the mapped reads, which together with TopHat2. The program reference annotation based transcripts (RABT) was preferred. Cufflinks constructed faux reads according to reference to make up for the influence of low coverage sequencing. During the last step of assembly, all of the reassembles fragments were aligned with reference genes and then similar fragments were removed.

Then we used Cuffmerge to merge transcripts from different replicas of a group into a comprehensive set of transcripts, and then merge the transcripts from multiple groups into a finally comprehensive set of transcripts for further downstream differential expression analysis.

1.5 Transcripts Identification

To identify the known and new transcripts, all of the reconstructed transcripts were aligned to reference genome and were divided into twelve categories by using Cuffcompare. We used the following parameters to identify reliable new transcripts:

1) Transcripts from unique mapped reads
2) Transcripts with one of the classcodes "u,i,j,x,c,e,o"
3) The length of transcript was longer than 200bp
4) The exon number was more than 2

1.6 LncRNA and Novel Protein-coding Transcripts Prediction

Three softwares Coding-Non-Coding-Index (CNCI) (version 2)[5], Coding Potential Calculator (CPC)[6] (http://cpc.cbi.pku.edu.cn/) and phylogenetic codon substitution frequency (PhyloCSF)[7] were used to assess the protein-coding potential of new transcripts by default parameters. The intersection of all results without protein-coding potential were chosen as long non-coding RNA transcripts. And the intersection of all results with protein-coding potential were chosen as novel protein-coding transcripts.

1.7 Alternative Splicing Analysis

The results of Tophat included all alternative splicing information and the junction structure is shown as follows:

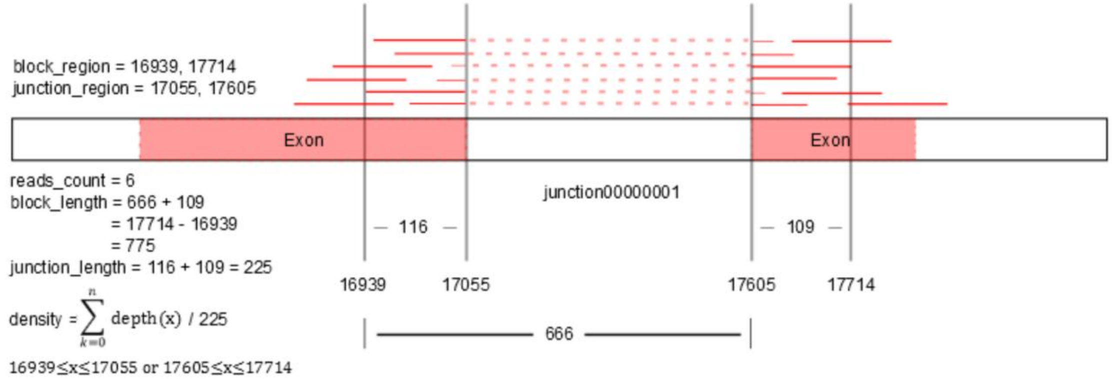

First the alternative splicing sites with less than 5 reads were filtered out. Then the alternative splicing sites were mapped to known alternative splicing sites (1bp error was allowable) to identify known alternative splicing sites. Finally, the unmapped new alternative splicing sites were classified.

The classification of alternative splicing is as follows:

I. Intergenic: Junctions start from and/or end up with the area between genes.

II. P5_splice: Junctions start inside an exon, end up with initiation site of another exon.
III. P3_splice: Junctions start from an exon termination site and end up inside another exon.
IV. ES: Junctions start from an exon termination site and end up with another termination site (the known alternative splicing was excluded).
V. IR: Junctions start and end in the same exon.
VI. AFS/TSS: Junctions start from the 5 'end of exon initiation site and end up with another exon.
VII. ALS/TTS: Junctions start from the 3’ end of the last exon initiation site and end up with another exon.
VIII. Others: Do not belong to the above situations

1.8 Quantification of Transcripts Abundance

Transcripts abundances were quantified by software RSEM[8]. There were two steps for RSEM to quantify transcripts abundances. Firstly, a set of reference transcript sequences were generated and preprocessed according to known transcripts and new transcripts and gene annotation files. Secondly, reads were realigned to the reference transcripts by Bowtie alignment program and the resulting alignments were used to estimate transcript abundances. The transcript expression level was normalized by using FPKM (Fragments Per Kilobase of transcript per Million mapped reads) method, and the formula is shown as follows:

$$\text{FPKM} = \frac{10^6 C}{NL/10^3}$$

Given FPKM(A) to be the expression of transcripts A, C to be number of fragments mapped to transcripts A, N to be total number of fragments that mapped to reference genes, and L to be number of bases on transcripts A. The FPKM method is able to eliminate the influence of different transcripts lengths and sequencing data amount on the calculation of transcripts expression. Therefore, the calculated transcripts expression can be directly used for comparing the difference of transcripts expression among samples.

1.9 Differentially expressed transcripts (DETs) Analysis

The differentially expressed transcripts of protein-coding and LncRNA transcripts were analyzed respectively. To identify differentially expressed transcripts across samples or groups, the edgeR package (http://www.r-project.org/) was used. We identified transcripts with a fold change ≥2 and a false discovery rate (FDR) <0.05 in a comparison as significant DETs. DETs were then

subjected to enrichment analysis of GO functions and KEGG pathways.

1.10 LncRNA cis-regulation Analysis

One of the functions of LncRNAs is cis-regulation of their neighboring genes on the same allele[9]. The up-stream LncRNAs which have intersection of promoter or other cis-elements may regulate gene expression in transcriptional or post-transcriptional level. The downstream or 3'UTR region LncRNAs may have other regulatory functions. Thus LncRNAs which had been previously annotated as "unknown region" were annotated again. LncRNAs in up/down stream of a gene were likely to be cis-regulators.

1.11 LncRNA trans-regulation Analysis

Another function of LncRNAs is trans-regulation of co-expressed genes not adjacent to LncRNAs[9]. We analysed the correlation of expression between LncRNA transcripts and protein-coding transcripts to identify target genes of LncRNAs.

2. miRNA analysis

2.1 Filtering of Clean Reads in small RNA-seq

The raw data were subjected to quality check using FastQC[1] (v0.11.4) (http://www.bioinformatics.babraham.ac.uk/projects/fastqc/). Reads obtained from the sequencing machines included dirty reads containing adapters or low quality bases which would affect the following assembly and analysis. Thus, to get clean reads, raw reads were further filtered according to the following rules:

1) Removing low quality reads containing more than one low quality (Q-value≤20) base or containing unknown nucleotides(N);

2) Removing reads without 3'adapters;

3) Removing reads containing 5'adapters;

4) Removing reads containing 3' and 5' adapters but no small RNA fragment between them;

5) Removing reads containing ployA in small RNA fragment;

6) Removing reads shorter than 18nt (not include adapters).

2.2 Alignment and Identification of small RNA

All of the clean reads were aligned with small RNAs in GeneBank database(Release 209.0) and to Rfam database(11.0) to identify and remove rRNA,scRNA,snoRNA,snRNA and tRNA. Meanwhile all of the clean reads were also aligned with reference genome. Those mapped to

exons or introns might be fragments from mRNA degradation, so these tags were removed. The reads mapped to repeat sequences were also removed[10].

The remaining clean reads were then searched against miRBase database(Release 21) to identify known miRNAs. All of the unannotated tags were aligned with reference genome. According to their genome positions and hairpin structures predicted by software Mireap_v0.2 [11], the novel miRNA candidates were identified. The default parameters of software Mireap_v0.2 were as follows:

1) Minimal miRNA sequence length is 18nt

2) Maximal miRNA sequence length is 26nt

3) Minimal miRNA reference sequence length is 20nt

4) Maximal miRNA reference sequence length is 24nt

5) Minimal depth of Drosha/Dicer cutting site is 3

6) Maximal copy number of miRNAs on reference is 20

7) Maximal free energy allowed for a miRNA precursor is 18 kcal/mol

8) Maximal space between miRNA and miRNA* is 35nt

9) Minimal space between miRNA and miRNA* is 14nt

10) Maximal bulge between miRNA and miRNA* is 4nt

11) Maximal asymmetry of miRNA/miRNA* duplex is 5nt

12) Flank sequence length of miRNA precursor is 10nt

2.3 miRNA expression profiles

The miRNA expression level was calculated and normalized to transcripts per million (TPM). The formula is as follows:

$$\text{TPM}=\text{Actual miRNA counts}/\text{Total counts of clean tags}*10^6$$

2.4 Differentially expressed miRNA (DE miRNA) Analysis

To identify differentially expressed miRNAs across samples or groups the formula was shown as follows:

$$p(x|y) = \left(\frac{N_2}{N_1}\right)^y \frac{(x+y)!}{x!\,y!\,(1+\frac{N_2}{N_1})^{(x+y+1)}} \qquad C(y \le y_{\min} | x) = \sum_{y=0}^{y \le y_{\min}} p(y|x) \qquad D(y \ge y_{\max} | x) = \sum_{y \ge y_{\max}}^{\infty} p(y|x)$$

We identified miRNAs with a fold change ≥2 and FDR <0.05 in a comparison as significant DE

miRNAs.

2.5 Target gene Prediction

Based on the sequences of the known miRNAs and novel miRNAs, three softwares RNAhybrid(v2.1.2)+svm_light(v6.01), Miranda(v3.3a) and TargetScan(Version:7.0) were used to predict target genes. The intersection of the results were more credible to be chosen as predicted miRNA target genes.

The default parameters of software RNAhybrid were as follows:

1) Forces structures to have a helix from position 2 to 8 with respect to the query

2) The number of hits per target is 1

3) Maximal bulge loop size is 3nt

4) Maximal internal loop size (per side) is 3nt

5) The cut-off P-value is 0.05

6) The cut-off energy is -10 kcal/mol

7) Maximal query length is 24nt

The default parameters of software Miranda were as follows:

1) The set score threshold is 140

2) The set energy threshold is -10 kcal/mol

3) Demand strict 5' seed pairing

4) The gap-open penalty is -4.0

5) The gap-extend penalty is -9.0

The default parameters of software TargetScan were as follows:

The 2-8nt sequences which start from 5' small RNA were choose as seed sequences to predict with 3'-UTR of protein-coding transcripts or full sequence of lncRNA transcripts.

References

1. Cock, P. J., Fields, C. J., Goto, N., Heuer, M. L. & Rice, P. M. The Sanger FASTQ file format for sequences with quality scores, and the Solexa/Illumina FASTQ variants. *Nucleic acids research* **38**, 1767-1771 (2009).
2. Langmead, B. & Salzberg, S. L. Fast gapped-read alignment with Bowtie 2. *Nature methods* **9**, 357-359 (2012).
3. Kim, D. *et al.* TopHat2: accurate alignment of transcriptomes in the presence of insertions, deletions and gene fusions. *Genome biology* **14**, 1 (2013).
4. Trapnell, C. *et al.* Differential gene and transcript expression analysis of RNA-seq experiments with TopHat and Cufflinks. *Nature protocols* **7**, 562-578 (2012).
5. Sun, L. *et al.* Utilizing sequence intrinsic composition to classify protein-coding and long non-coding transcripts. *Nucleic acids research*, gkt646 (2013).
6. Kong, L. *et al.* CPC: assess the protein-coding potential of transcripts using sequence features and support vector machine. *Nucleic acids research* **35**, W345-W349 (2007).

7. Zhan, S. *et al.* Genome-wide identification and characterization of long non-coding RNAs in developmental skeletal muscle of fetal goat. *BMC genomics* **17**, 666 (2016).
8. Li, B. & Dewey, C. N. RSEM: accurate transcript quantification from RNA-Seq data with or without a reference genome. *BMC bioinformatics* **12**, 1 (2011).
9. Han, D. *et al.* Transcriptome analyses of differential gene expression in the bursa of Fabricius between Silky Fowl and White Leghorn. *Scientific reports* **7** (2017).
10. Langmead, B., Trapnell, C., Pop, M. & Salzberg, S. L. Ultrafast and memory-efficient alignment of short DNA sequences to the human genome. *Genome biology* **10**, 1 (2009).
11. Hafner, M. *et al.* Identification of microRNAs and other small regulatory RNAs using cDNA library sequencing. *Methods* **44**, 3-12 (2008).
